# Modeling Eye Movements During Decision Making: A Review

**DOI:** 10.1007/s11336-022-09876-4

**Published:** 2022-07-19

**Authors:** Michel Wedel, Rik Pieters, Ralf van der Lans

**Affiliations:** 1 Robert H. Smith School of Business, University of Maryland, College Park, MD 20742-1815 USA; 2 Tilburg University, Tilburg, The Netherlands; 3 Católica Lisbon School of Business and Economics, Universidade Católica Portuguesa, Lisbon, Portugal; 4 Hong Kong University of Science and Technology, Clear Water Bay, Kowloon, Hong Kong

**Keywords:** eye movements, task switching, choice, search, decision making, hidden Markov model

## Abstract

This article reviews recent advances in the psychometric and econometric modeling of eye-movements during decision making. Eye movements offer a unique window on unobserved perceptual, cognitive, and evaluative processes of people who are engaged in decision making tasks. They provide new insights into these processes, which are not easily available otherwise, allow for explanations of fundamental search and choice phenomena, and enable predictions of future decisions. We propose a theoretical framework of the search and choice tasks that people commonly engage in and of the underlying cognitive processes involved in those tasks. We discuss how these processes drive specific eye-movement patterns. Our framework emphasizes the central role of task and strategy switching for complex goal attainment. We place the extant literature within that framework, highlight recent advances in modeling eye-movement behaviors during search and choice, discuss limitations, challenges, and open problems. An agenda for further psychometric modeling of eye movements during decision making concludes the review.

People make hundreds of decisions each day. For instance, car drivers search for traffic signs and decide to slow down or yield right-of-way (Ho, Scialfa, Caird, & Graw, 2001), airport security personnel search for weapons in X-ray images of luggage and decide whether to do a physical examination (McCarley, Kramer, Wickens, Vidoni, & Boot, 2004), radiologists search for nodules on mammograms and chest radiographs and decide which ones are potentially cancerous (Krupinski, Berger, Dallas, & Roehrig, 2003; Wedel, Yan, Siegel, & Li, 2016), people look at facial characteristics to decide which person they like (Chuk, Chan, Shimojo, & Hsiao, 2020), consumers search for products on websites and shelves (Shi, Wedel, & Pieters, 2013; van der Lans, Pieters, & Wedel, 2008b), search for information about prices, ingredients, and sustainability on packages (van Herpen & van Trijp, 2011), and choose the products they expect to satisfy their needs (Stüttgen, Boatwright, & Monroe, 2012). All these decisions involve search as well as choice. The underlying cognitive mechanisms rely heavily on acquisition and processing of visual information.

There is a long history of research into search and choice behaviors in psychology and related disciplines (examples are Berlyne, 1971; Russo & Rosen, 1975; Wolfe, 1998; Yarbus, 1967). This research has yielded a deep understanding of the underlying fundamental processes. Yet, three challenges face this literature. First, although many forms of decision making involve visual search, search and choice have been addressed in disparate streams of research, which has resulted in suboptimal cross-fertilization of knowledge. Second, much of the research in question has relied on endpoint measures, such as response times and the final choices made, to infer the underlying cognitive mechanisms of interest (Luce, 1977; McFadden, 1974; Treisman & Gelade, 1980; Wolfe, 1998). Such endpoint measures have proven to be valuable, but do not always provide unambiguous evidence for the underlying processes (Sanders & Donk, 1996; Zelinsky, 2008). Third, extant research has often relied on simple stimuli and controlled tasks in high-repetition, within-participant designs to uniquely identify the processes of interest. These controlled experiments ensure a high internal validity of hypothesis tests, yet may suffer from limited ecological validity of inferences on the cognitive processes as they play out in real-life contexts.

There is growing interest in understanding search and choice in the common, more complex, messy, and less controlled tasks that people encounter in their daily lives. Such research faces critical challenges because search and choice behaviors are often involved in the same task, and multiple unobserved cognitive processes may simultaneously cause variations in the associated endpoint measures. Eye movements are unique process measures to study search and choice behavior in such natural contexts (Findlay & Gilchrist, 2003; Najemnik & Geisler, 2005; Zelinsky, 2008). Eye movements reflect, with a high temporal and spatial resolution, several unobserved perceptual, cognitive and evaluative processes (Findlay & Gilchrist, 1998). They enable a fine-grained process analysis with the potential of yielding insights that are difficult to obtain otherwise, especially in real-life contexts. For example, eye movements have provided insights into information search during advertising exposure (Wedel & Pieters, 2000) and brand choice (Shi et al., 2013), the determinant processes of which are largely inaccessible to self-reports (Aribarg, Pieters, & Wedel, 2010; Nisbett & Wilson, 1977) or to alternative process-tracing techniques such as information display boards (Lohse & Johnson, 1996). Statistical models have proven to be indispensable for making inferences on these unobservable processes, in psychology, economics and marketing, because they enable disentangling the effects of multiple unobserved processes from the eye-movement recordings (Stüttgen et al., 2012; van der Lans et al., 2008b; Yang, Toubia, & de Jong, 2015). Understanding the mechanisms that drive search and choice may lead to better predictions and has ramifications for policy makers, companies, and consumers, for example by enabling better design and testing of policy and medical interventions, websites, store shelves, advertisements, and labels on food packaging (e.g., Van Loo, Grebitus, Nayga Jr., Verbeke, & Roosen, 2018).

Several streams of research have examined search and choice behaviors in natural environments by using eye-movement measures and statistical, psychometric, or econometric models. Earlier reviews have summarized advances in eye-tracking research, in general (Rayner, 1998; Wedel & Pieters, 2008a) and for search and choice separately (Glaholt & Reingold, 2011; Kowler, 2011; Orquin & Loose, 2013), or have conducted meta-analyses of eye movements during choice (Orquin, Lahm, & Stojić, 2021). The present article reviews the advances made in the modeling of eye movements during decision making. We aim to make a step toward an integrated account of the cognitive processes fundamental to decision making by highlighting the role of task and strategy switching during such decision making. We provide a theoretical framework that summarizes the key tasks involved and the processes underlying eye movements during decision making. We place the extant literature within that framework, highlight recent advances in modeling eye-movement behaviors, discuss limitations, challenges, and open problems, and provide an agenda for future research. We believe that such a review of the literature is timely, because the cost of eye-tracking equipment has come down enormously and relatively unobtrusive eye-movement recording is now possible in a wide variety of real-life contexts, while the psychometric toolbox to extract information from eye-tracking data is rapidly growing. Even so, eye-movement recording and modeling during search and choice is still underutilized both in academic and in applied research, a situation which we hope this review will help to improve.

Section 1 briefly introduces eye movements and eye-movement recording. Section 2 presents our framework of tasks, task switching, and attentional processes underlying eye movements during decision making. Section 3 summarizes models for each of those processes during search. Section 4 reviews, against this backdrop, the literature on eye-movement analysis during choice. Section 5 provides an outlook for novel applications and future developments.

## Eye Movements and Their Recording

### Eye Movements and Visual Attention

We use the term (visual) attention as a container for various cognitive processes that are involved in the selection of relevant and the suppression of irrelevant information during search and choice, for which according to James (1890, pp. 403–404): “Focalization, concentration, and consciousness are of […] essence.” The next section describes these processes and their links to eye movements. It is important to point out that in most natural decision-making tasks, eye-movement patterns are valid and accurate indicators of the hidden attention processes of interest (Findlay, 2005). There are also successful examples of inferences on higher order cognitive states from eye movements, such as on tasks and goals (Borji & Itti, 2014; Haji-Abolhassani & Clark, 2014; Kardan, Berman, Yourganov, Schmidt, & Henderson, 2015) and on the emotional valence of images (Nummenmaa, Hyönä, & Calvo, 2006; R.-Tavakoli et al., 2015), although these tend to require additional experimental control, measures, and theoretical assumptions.

At any point in time, the human eye-vision system processes only about one percent of the field of vision with high acuity (Rayner, 1998; Wedel & Pieters, 2008a). This is due to the density of photosensitive cells in the human retina decreasing rapidly from a high concentration in a small region in its center (fovea) toward the parafovea and periphery. Therefore, people need to move their eyes to explore their environment in sufficient detail. In practice, people make on average three to six larger eye movements per second during tasks such as reading, scene perception, or decision making (Rayner, 1998). These saccades are fast jumps of the eye’s point of regard (POR) between spatial locations in the environment. Saccades typically last for about 20–40 ms. Fixations are periods of about 100–400 ms when the eyes hardly move. While during a saccade visual perception is suppressed to prevent blurring, during an eye fixation detailed visual information is obtained from a small region of about two degrees of visual angle around the POR.

**Fig. 1:**
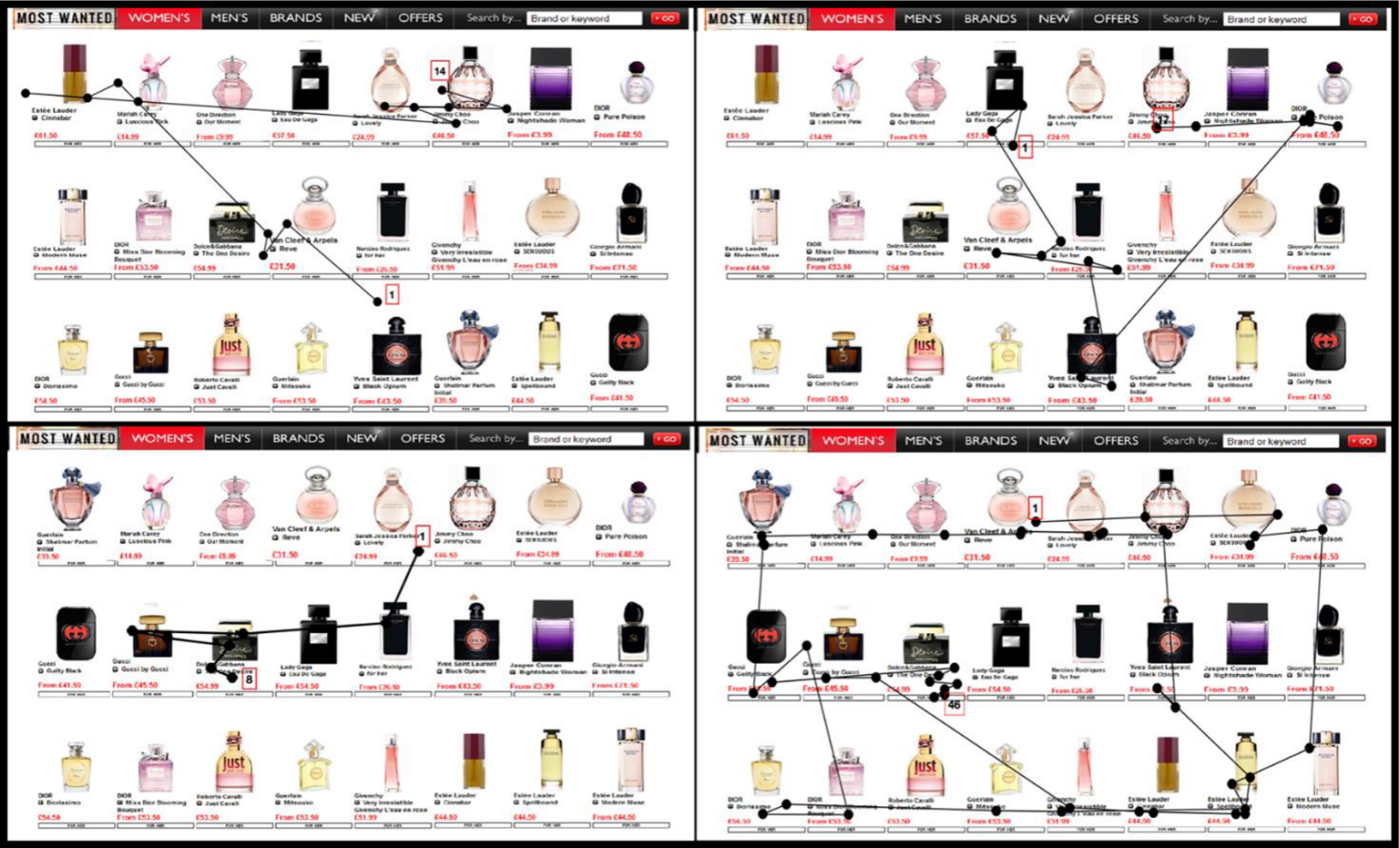
Scan-paths of four participants searching for a brand of perfume to choose on a shopping website. The dots indicate fixations, the lines saccades. Red rectangular boxes indicate the first and last fixation. The top, respectively, bottom, panels show scan-paths for two participants searching for the Jimmy Choo, respectively, the Dolce & Gabana, perfumes (based on data from van der Lans et al., 2021).

A scan-path is the spatiotemporal sequence of eye fixations and saccades for a particular task, stimulus, and person. Figure 1 summarizes the scan-paths of four participants who were engaged in a target search task: they searched on a shopping website for a brand of perfume to choose as a gift. The figure (based on data from study 2 in van der Lans, Pieters, & Wedel, 2021) reveals several key features of the scan-paths which inform theories and psychometric models. First, the first fixation commonly lands close to the center of the screen. This location may be optimal because it has the smallest distance to all other locations. Second, some fixations land on visually salient objects, i.e., objects that stand out from their surroundings in terms of luminance, color, or shape. Other parts of individuals’ scan-paths reflect systematic left-right movements. That pattern may be induced by the organization of the image (scene layout). Third, the scan-paths consist of clusters of fixations on objects, interspersed with saccades toward more distant objects. Those clusters reflect refixations which are used to accumulate information to determine the identity or value of objects of interest. Longer saccades reflect search for where new objects are located. Fourth, a cluster of fixations is specifically directed at the final, chosen object. Such a pattern may reflect preference formation or a final verification of the object before implementing a decision, or it might be that looking at an object longer increases one’s preference for it. Fifth, scan-paths show large individual variation and differ markedly among distinct tasks and stimuli. In addition, the scan-paths of participants who had a different search goal (in the top versus the bottom row of the figure) are noticeably different. We return to all those features of scan-paths in the sequel. The scan-paths observed when people make choices among alternative options are similar to those in Fig. 1 but are often considerably more elaborate.

Next to fixations and saccades, other types of eye movements include micro-saccades which are involuntary movements with small amplitude that occur during fixations, smooth pursuits which are fixations on moving objects, vergence movements which occur to maintain the POR on an object that moves toward or away from the viewer, and pupil movements to regulate the amount of incoming light, amongst others. While the analysis of these movements has been useful for specific problems, the present review focuses on fixations and saccades.

### Eye-Movement Recording

The dominant method of recording eye movements in research on search and choice uses an infrared light source directed at one or both eyes, which creates (invisible) reflections on the cornea, the hard outer layer of each eye, called Purkinje reflections (Duchowski, 2003). After a short calibration task, video cameras record one or more of these reflections, even while participants move their heads. Based on the calibration results, algorithms infer the POR in x-y stimulus coordinates from these reflections. Commonly used eye trackers are built into desktop monitors or into small stand-alone devices that can be attached to laptops and other objects. In addition, mobile eye trackers embedded in glasses allow free body movements while recording the entire field of view as well as the POR within it. Commercial infrared eye trackers typically sample the POR with a sampling frequency of 50 or 60 Hertz and a spatial accuracy of 0.5 degrees of visual angle, or better. Eye trackers with a higher spatiotemporal precision make use of multiple Purkinje reflections and sampling frequencies of 120 Hertz or more, for which the participant’s head sometimes needs to be fixed during recording with a chin or forehead rest. Further, eye movements can be recorded with regular front-facing (web) cameras integrated into digital devices, by using eye landmarks, such as the center and shape of the pupil and the location of eye corners, as input to computer vision algorithms that estimate gaze direction from images of the eyes. Those eye-tracking solutions still have lower accuracy than infrared eye tracking.

### Eye-Movement Data

Eye tracking during decision making results in large amounts of raw data. For example, an eye-tracking study in which participants make 20 decisions, with 10 s available for each decision and a 50 Hertz sampling frequency, results in some ten thousand samples comprised of the x-y coordinates of the POR of each eye per participant. This amounts to one million data points for a study with one hundred participants. A proportion of the data may involve outliers or may be missing due to eye blinks and tracking problems, however. From the raw samples of the POR, algorithms are used to identify fixations and saccades based on their spatial proximity or the velocity of their movement (Salvucci & Goldberg, 2000). This reduces the dimensionality of the data by a factor 20 or more. For example, the BIT (Binocular Individual Threshold) algorithm (van der Lans, Wedel, & Pieters, 2011) automatically determines fixations using individual-specific eye-velocity thresholds for both eyes, automatically removing eye blinks and other recording abnormalities. Due to blinks, saccades and recording errors, missing data points are common in raw samples of the POR. As a rule of thumb, van der Lans and Wedel (2017) propose that one may consider removing a participant’s data if less than 80 percent of their raw samples are classified as fixations. Once the fixation data have been prepared, various characteristics of the eye-movement patterns can be examined, such as x-y coordinates of fixations over time, scan-paths, or aggregate measures such as fixation counts, gaze or dwell times, and fixation selection indicators, on so-called Regions of Interest (ROIs) (Holmqvist et al., 2011). The spatial aggregation of fixations on those ROIs, for example by calculating gaze times on the individual perfume bottles in Fig. 1, reduces the size of the data further (the gaze time on a ROI is defined as the sum of the durations of all fixations that land on that ROI). Fixation patterns are often graphically explored via heatmaps, which represent the density of fixations using colors, and fixation plots which depict scan-paths as shown in Fig. 1.

## Theoretical Framework

Via eye movements, people acquire information to reduce various uncertainties during task completion (Hayhoe & Ballard, 2005; Land, 2019; Pieters & Wedel, 2007). Decision-making tasks vary in the types of uncertainty that they induce, which influences the eye-movement patterns observed during these tasks. We propose a classification of decision-making tasks in Sect. 2.1 and, building on this, a theoretical framework of eye movements during decision making in Sect. 2.2.

### Decision-Making Tasks

In Table 1, we propose a classification that involves six fundamental decision-making tasks that people commonly engage in. These tasks have in common that they require eye movements. They therefore involve exposure durations longer than the duration of a single fixation (100–300 ms). The tasks elicit different types of uncertainties which people try to resolve through visual information acquisition.

**Table 1:** Six fundamental decision-making tasks, the types of uncertainty they induce, and the dominant cognitive process involved when engaging in the task.

I. Task		II. Uncertainty	III. Example	IV. Dominant Process
Perceptual Decision Making: Search				
1	Localization	Space	Where in the scene is object j, if at all?	Perception
2	Identification	Object	Is this object j or not, or is it object k?	Perception
3	Specification	Attribute	Does object j have attribute m, and how much of it?	Cognition
Preferential Decision Making: Choice				
4	Inference	Outcome	Will attribute m of object j lead to outcome y for person i?	Cognition
5	Evaluation	Value	How valuable or meaningful is attribute m (of object j) to person i?	Evaluation
6	Integration	Utility	How much does person i value object j (over object k)?	Evaluation

Table 1 makes a main distinction between *perceptual decision making *and *preferential decision making* (e.g., Dutilh & Rieskamp, 2016; Summerfield & Blangero, 2017). Perceptual decision-making tasks have an objective (external) performance criterion, mostly speed and/or accuracy. Search tasks are perceptual decision-making tasks. Preferential (or value-based) decision-making tasks have one or more subjective (internal) performance criteria, such as perceived costs and benefits, decision justification, or post-choice satisfaction. The completion of these tasks is based on personal preference or utility functions. Choice tasks are preferential decision-making tasks. The dominant processes involved in the tasks in Table 1 are perception, cognition, and evaluation, respectively (Table 1, column IV). Although cognition is sometimes used in a broader sense to encompass perception and evaluation, here *perception* refers to the process of acquiring new information via the senses, *cognition* refers to the processes of storing, retrieving, interpreting, and combining new and existing information, and *evaluation* refers to assessing the personal value of the information (and acting upon it).

We distinguish six basic tasks, each having its own specific type of uncertainty that people need to reduce (Table 1, columns I and II), and each being at least partly observable from eye movements. 1. *Localization* aims to reduce uncertainty about the spatial location of a known object; 2. *Identification* aims to reduce uncertainty about the identity of an object vis-à-vis other objects and/or some object template in memory; 3. *Specification* aims to reduce uncertainty about the presence of specific attributes or features of an object by associating the object with information on these attributes in memory; 4. *Inference* aims to reduce uncertainty about the consequences of these attributes and their decision outcomes; 5. *Valuation* aims to reduce uncertainty about the value or meaningfulness of an object’s attributes and features to the person’s goals; 6. *Integration* aims to reduce uncertainty about the overall utility of an object (choice alternative) to the person relative to other objects. We use the term “integration,” because people need to integrate expected outcomes and their valuation of these outcomes into their overall assessment of the utility of a stimulus (Anderson & Shanteau, 1970). Integration can take place via some (optimal) subjective expected utility mechanism, as traditionally assumed in economics, or via satisficing mechanisms (Simon, 1955). The order of uncertainties in the table from top to bottom corresponds to the hierarchical organization of knowledge structures from bottom-up features of objects to top-down values and preferences of people. It reflects the structure of the human motivation system from concrete means (objects and their attributes), which have expected consequences (outcomes), which satisfy certain abstract ends (values) (Kruglanski, Chernikova, Babush, Dugas, & Schumpe, 2015), which contribute to utility.

The tasks that people engage in in real-life contexts are commonly combinations of these six basic tasks. For example, localization (task 1) and identification (task 2) are subtasks of a target search task (van der Lans et al., 2008b). Localization (task 1) and specification (task 3) are subtasks of an information search task (Moorthy, Ratchford, & Talukdar, 1997). Identification (task 2) and specification (task 3) are subtasks of a categorization task (Rosch, 1978), in which people differentiate prototypes from exemplars in a category or exemplars in one category from exemplars in another. It is crucial to note that whereas controlled experiments can be used to isolate a single task or uncertainty, the tasks that people perform in natural settings are mostly comprised of several subtasks, each with their specific uncertainty (Droll & Hayhoe, 2007; Hayhoe & Ballard, 2005; Locke & Latham, 2002).

### Theoretical Framework

**Fig. 2:**
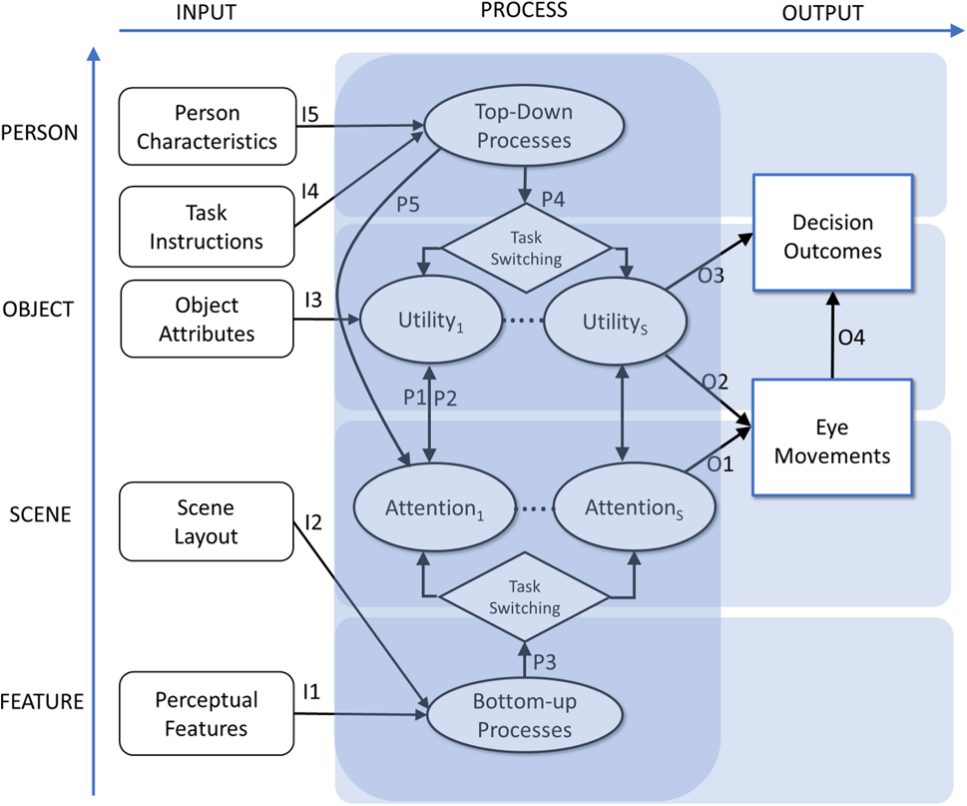
Eye movements and decision task performance. Framework for how people use eye movements to reduce uncertainties inherent in perceptual and preferential decision-making tasks. Observable exogenous inputs to the decision processes are on the left side; unobservable perceptual, cognitive, and evaluative processes are in the center; observable decision outcomes are on the right-side. Inputs and processes are organized from top to bottom by level of visual processing: person, object, scene, and feature. Arrows depict the links between inputs and processes (I1 to I5), between unobserved processes (P1 to P5), and between processes and decision outputs (O1 to O4).

The classification in Table 1 informs our framework, summarized in Fig. 2, of how people use eye movements to reduce uncertainties inherent in perceptual and preferential decision-making tasks. The framework relates eye movements to the underlying processes of interest. It shows from left to right 1) inputs, 2) processes, and 3) decision outputs. These concepts are organized from bottom to top by level of processing, from features, to scene, objects, and the person. Symbols indicate the links between these concepts.

First, the framework distinguishes five observable exogenous inputs: a) person characteristics (i.e., age, prior knowledge), b) task instructions (i.e., search goal, incentives, time constraints), c) object attributes (i.e., description of objects/products on dimensions such as price, brand name and other qualities), d) scene layout (i.e., how the objects are arranged on a row-column shopping website), and e) perceptual features (i.e., detailed visual characteristics, such as colors and edges of objects). Second, the outputs of decision tasks consist of observable micro- (fixation locations, fixation durations) and macro-decision outcomes (search accuracy, choice, and response time), which are represented at the right-hand side of Fig. 2. Third, in between these observable inputs and outputs are unobserved, or latent, processes, depicted in the center of the figure (connected via P1-P5). These latent processes are affected by exogenous inputs (via links I1-I5), and in turn impact both eye movement (via O1-O2) and choice decisions (via O3-O4).

Task and strategy switching take a central role in the framework. Formally, task switching concerns switching between two or more tasks which each have their own unique goals and uncertainties (Kiesel et al., 2010), whereas strategy switching concerns switching between different ways of pursuing the same task or goal (Shi et al., 2013). In this review, we use the terms interchangeably, because in real-life contexts, empirically the distinction between task and strategy switching is often ambiguous. Decision making in real-life contexts typically involves the deployment of attention to resolve multiple uncertainties over time (Table 1). Therefore, people break up complex decision tasks into multiple simpler subtasks, each of which requires a unique strategy that involves the deployment of attention to resolve the associated uncertainty (Stewart, Hermens, & Matthews, 2016; Stojić, Orquin, Dayan, Dolan, & Speekenbrink, 2020). People switch between these subtasks over time to assess the utility of decision alternatives and meet the overall decision goal, under cognitive cost and effort constraints. They monitor goal progress within tasks, and switch when a task nears completion and/or incremental expected utility drops below or cost rises above a threshold (Gutzwiller, Wickens, & Clegg, 2019). Such task/strategy switching characterizes goal pursuit and decision making in complex, realistic contexts (Locke & Latham, 2002). The next sections describe the attention processes during decision making. Appendix 1 summarizes the neuroscientific basis of attention and eye movements.

#### Eye Movements and Attention

Overt (observable) eye movements are tightly coupled with covert (unobservable) shifts of visual attention (Fig. 2, O1). Attention and eye movements are guided by the same regions in the brain (Corbetta & Shulman, 2002; Findlay, 2005; Findlay & Gilchrist, 2003; Appendix 1), and their coupling has been likened to a rubber band (Henderson & Hollingworth, 1998): “the eyes go where attention goes and attention goes where the eyes go”. Therefore, and because they are subject to (mechanical) measurement errors (Duchowski, 2003), eye-movement recordings are probabilistic indicators of the location and duration of covert attention (Baddeley & Tatler, 2006).

#### Task and Strategy Switching

Most real-life decision tasks require the reduction in more than one type of uncertainty (Table 1). Rather than reducing these uncertainties simultaneously, during real-life decision making people switch between attention/decision strategies that each aim at reducing a specific type of uncertainty (Haji-Abolhassani & Clark, 2013, 2014; Liechty, Pieters, & Wedel, 2003; van der Lans, Pieters, & Wedel, 2008a; van der Lans et al., 2008b; Wedel, Pieters, & Liechty, 2008). Because the human visual system uses eye movements to acquire information “just in time” when the demands of the current task call for it (Hayhoe & Ballard, 2005), shifts in these strategies are reflected in the observed scan-path of eye movements (Mayr, Kuhns, & Rieter, 2013).

Strategy shifts may occur because of competition between subtasks. Task schemas that drive attentional processes may exert mutually inhibitory effects (Gilbert & Shallice, 2002). Top-down (executive) control may also be involved to selectively activate or deactivate these schemas, depending on which subtask has gained priority (Logan & Gordon, 2001). Such subconscious task interference and volitional top-down control may both operate at the same time (Kiesel et al., 2010). Strategy switching is thus central to decision making in complex real-life settings (Venkatraman, Payne, & Huettel, 2014). We submit that it is necessary for implementing both micro- (eye movement; Fig. 2, O1) and macro-(search and choice; Fig. 2, O3) decisions that facilitate the attainment of the overall task goal. Instances of attentional strategy switching are (1) processing the location versus the identity of objects during target search (van der Lans et al., 2008a; b), (2) processing by object across attributes versus by attribute across objects during information search and choice (Shi et al., 2013), (3) systematic versus salience-based attention allocation during target search (van der Lans et al., 2008a; b), and (4) habitual versus goal-directed processing during choice (Ursu, Zhang, & Erdem, 2021). To illustrate, we expand on the first two of these processes, which occur during perceptual (location/identity) and preferential (by attribute/object) decision making, respectively. Sections 3.1 and 3.2 describe these processes in more detail.

First, during perceptual decision making, visual information processing in the human brain takes place in two broad pathways (Glaholt & Reingold, 2011; Ungerleider & Mishkin, 1982; Appendix 1), each tailored to reduce a specific type of uncertainty (Table 1). The “what” pathway is involved in identification of objects (faces, hands, people, houses). The “where” pathway is involved in the localization of objects. The “what” (*identification*) and “where” (*localization*) pathways give rise to different oculomotor patterns (Bullier, Schall, & Morel, 1996; Pannasch & Velichkovsky, 2009; Appendix 1). Activity in the “what” stream tends to produce short saccades (Liechty et al., 2003; Pannasch & Velichkovsky, 2009), with fixations that cluster in a small number of regions that are deemed informative to the task (Smith & Henderson, 2009; Wedel et al., 2008; Yarbus, 1967). Those short saccades result in repeated fixations on an object, which minimizes working memory load and may be required for target identification and specification of the object in terms of its attributes (Droll & Hayhoe, 2007; Rayner, Smith, Malcolm, & Henderson, 2009; Smith & Henderson, 2009). Tasks with more complex stimuli (naturalistic versus abstract stimuli) and a higher working memory load (choice versus search tasks) therefore typically result in more and longer fixations (Gould, 1973; Orquin & Loose, 2013). Activity of the “where” stream tends to produce longer saccades that serve to rapidly bring peripheral, salient locations and objects into focus. Eye-tracking research has revealed that people frequently switch back and forth between such “what” and “where” strategies, which, respectively, involve repeated fixations on a small region, and longer saccades between distant regions (Liechty et al., 2003; Pannasch & Velichkovsky, 2009; Wedel et al., 2008).

As a second example, during preferential decision making on attribute-by-product matrices, people acquire information using *processing-by-attribute* or *processing-by-product* strategies (Bettman, Luce, & Payne, 1998; Payne, Bettman, & Johnson, 1993). Attribute-based processing involves extraction of information on a single attribute across multiple products. Such processing involves a low-effort attribute-specification strategy that serves to compare products. Product-based processing involves acquisition of information on a single product across multiple attributes. It is a high-effort strategy which supports the integration of information into an overall expected value or utility (Martinovici, Pieters, & Erdem, 2021). Early process-tracing methods such as Mouselab, where participants open and close information cells on an electronic display, suggested that people first use by-attribute processing and then switch to by-product processing before making a choice (Bettman et al., 1998). Eye-tracking research initially confirmed that during preferential decisions people first tend to make more inter-product saccades which reflect processing-by-attribute, and then more intra-product saccades which reflect a processing-by-product strategy (Pieters & Warlop, 1999). However, rather than reflecting a simple and orderly two-stage process, eye-movement data have demonstrated that people repeatedly switch between these two strategies (Table 1) even during decision-making tasks that last a few minutes at most. People tend to extract information on two or three products in a by-attribute strategy, and on two or three attributes in a by-product strategy, and switch back and forth between these two strategies multiple times before making a choice (Shi et al., 2013).

#### Bottom–Up Factors

Bottom-up factors residing in the stimulus affect attention processes and thus eye movements (Fig. 2, P3), and exert a large influence especially during object localization. These visual factors have strong effects on attention, comparable in size to those of top-down factors such as task instructions (Orquin et al., 2021). Basic perceptual features, such as luminance, edges, contours, and colors (Treisman & Gelade, 1980; Wolfe, 1994), are extracted and combined into a *salience map*, or attention priority map (Fig. 2, I1**; **Appendix 1) (Itti & Koch, 2001; Itti, Koch, & Niebur, 1998; Koch & Ullman, 1985). The attention priority map represents the conspicuousness of locations in the visual field and guides attention and eye movements (Fig. 2, P3) (Donk & Soesman, 2010; Treisman & Gelade, 1980), because the focus of attention successively shifts to locations on the map with decreasing priority (Foulsham & Underwood, 2008; Parkhurst, Law, & Niebur, 2002). Perceptual *pop-out* occurs when a particular location in the visual field stands out due to a basic feature that draws attention almost immediately (Parkhurst et al., 2002; Treisman & Gelade, 1980). *Inhibition of return* (IOR; Klein, 2000; Posner & Cohen, 1984) encourages exploration of novel locations by temporarily inhibiting the return of the eyes to previously attended locations or objects (Appendix 1).

The global layout of the scene also influences eye movements via what is called *contextual guidance* (Fig. 2, I2) (Torralba, Oliva, Castelhano, & Henderson, 2006). The essential meaning of a scene, or its gist, is extracted bottom up based on the spatial distribution of low-level features (Oliva & Schyns, 2000; Oliva & Torralba, 2006). This process is fast. The gist of a typical scene can be perceived even within a single fixation, in less than 100 msec. (Pieters & Wedel, 2012; Rousselet, Joubert, & Fabre-Thorpe, 2005), presumably immediately upon exposure to it. Gist perception helps the localization and identification of objects and guides the initial scan-path (Mack, Gauthier, Sadr, & Palmeri, 2008; Appendix 1).

During search tasks on visual scenes, fixating on the center of the scene first may be optimal for assessing the gist and basic features across the entire visual field (Itti & Koch, 2001; Koch & Ullman, 1985). This optimal viewing position facilitates subsequent eye movements to salient or informative locations (Tatler, 2007). Therefore, there is a tendency to begin the scan-path close to the center of the image or computer screen on which the scene, for example an advertisement or shopping website (Fig. 1), is presented (Mannan, Ruddock, & Wooding, 1995; Parkhurst & Niebur, 2003; Reinagel & Zador, 1999; Tatler, 2007). More generally, the layout of visual displays has been shown to affect information acquisition processes and the direction of the scan-path (Glaholt, Wu, & Reingold, 2010; Pieters & Warlop, 1999; Shi et al., 2013; Fig. 2, I2).

#### Top–Down Factors

Top-down factors, residing in the person and the task, such as memory (Olivers, Meijer, & Theeuwes, 2006) and goals (Hayhoe & Ballard, 2005; Pieters & Wedel, 2020), affect eye movements in an interplay with bottom-up processes (Fig. 2, P4, P5). Yarbus (1967) first showed how eye movements are dramatically directed toward stimuli that are informative for the current task or goal. Attention to visual features the viewer believes to be instrumental for task performance is enhanced, and attention to features that are deemed irrelevant is suppressed, via top-down influences on the attention priority map (Pieters & Wedel, 2007; Fig. 2, P5; Appendix 1).

Top-down processes may also be primed by the global layout of a visual scene, which may lead to orderly spatial sequences of eye movements that reflect individuals’ use of systematic search and processing strategies (Monk, 1984; Ponsoda, Scott, & Findlay, 1995; Spalek & Hammad, 2005). These occur, for example, when a natural scene with a horizontal layout of objects or text primes the first fixation to be at the top-left, and the saccades to be predominantly from left to right (Shi et al., 2013).

Practice and time pressure are also key top-down factors (Fig. 2, I4). Practice improves decision performance by making the inhibition of distracting stimuli more efficient. Practice results in (1) improved speed of locating and discriminating targets from distractors, i.e., in faster reduction in location and identity uncertainty, and in (2) shorter fixation durations and thus faster response times (response times during decision tasks are approximately equal to the sum of all fixation durations; van der Lans et al., 2021; Zelinsky & Sheinberg, 1997). When making decisions under time pressure, people switch from more complete full-information search strategies to more efficient partial-information search strategies (Pieters & Warlop, 1999) which may involve (1) shortening fixation durations, or (2) filtering a subset of the available information by ignoring certain objects or their attributes, or (3) shifting to a different acquisition strategy all together (Fig. 2, P4). Specifically, people may switch from a processing-by-product, characterized by intra-product saccades, to a processing-by-attribute strategy, characterized by inter-product saccades. Individual differences in eye movements may be due to a variety of unobservable states and traits and have been documented in reading, scene viewing, and various types of decision tasks (Fig. 2, I5; e.g., Henderson & Hollingworth, 1998; Lee & Webb, 2005; Pieters & Wedel, 2007; Rayner, 2009; Rutishauser & Koch, 2007; Shen & Palmeri, 2016).

#### Utility/Value

Utility, or subjective value, is a measure of the happiness or satisfaction that consumers get from searching, acquiring and/or experiencing goods and services. Economic decisions are often assumed to be maximizing utility: the best choice outcome provides the highest expected utility to the decision maker (McFadden, 1974). Several dual-system accounts of decision making have been proposed for value learning and value-based decision making (Damasio, 1994; Daw & O’Doherty, 2014; Kahneman, 2011; Wang, 2002), whereby reflexive versus reflective decisions, compensatory versus satisficing decisions, or decisions pertaining to positive versus negative departures from a baseline, are controlled by mutually inhibiting (top-down) systems (Fig. 2, P4).

The empirical association between eye-movement measures of visual attention and value or choice outcomes is well established (Isham & Geng, 2013; Krajbich, Armel, & Rangel, 2010; Pieters & Warlop, 1999; Schotter, Berry, McKenzie, & Rayner, 2010; Shi et al., 2013; Shimojo, Simion, Shimojo, & Scheier, 2003; Stewart et al., 2016; Stojić et al., 2020). Evidence for the role of attention in choice comes from studies that use statistical mediation analysis to assess the extent to which the effect of visual factors on choice behavior is mediated via visual attention, by calculating their indirect effects through the mediator (Zhang, Wedel, & Pieters, 2009). Those studies have looked into the effects of visual marketing factors such as package design (Milosavljevic, Navalpakkam, Koch, & Rangel, 2012), nutrition information (Bialkova & van Trijp, 2011), shelf position (Atalay, Bodur, & Rasolofoarison, 2012; Chandon, Hutchinson, Bradlow, & Young, 2009; Chen, Burke, Hui, & Leykin, 2021; Deng, Kahn, Unnava, & Lee, 2016), assortment (Townsend & Kahn, 2013), advertisement displays (Zhang et al., 2009) and product salience on websites (van der Lans et al., 2021). This stream of research has provided consistent evidence that the effects of visual factors on search and choice outcomes are statistically mediated by eye-movement metrics.

One significant top-down effect occurs when a previously rewarded stimulus continues to capture attention automatically even if it is no longer associated with the reward (Fig. 2, P5) (Anderson, Laurent, & Yantis, 2011; Della Libera & Chelazzi, 2006). The learned stimulus-reward association results in an enduring attentional priority and a very slow extinction response (Anderson & Yantis, 2013) that hampers suppression of that stimulus during subsequent search and choice for task-relevant stimuli.

Importantly, the relationship between utility and attention is bidirectional (Fig. 1, P1 P2). Despite some alternative accounts (Glaholt & Reingold, 2011), there is converging evidence that people tend to look more at what they like (Fig. 2, P1; Callaway, Rangel, & Griffiths, 2021; Gluth, Kern, Kortmann, & Vitali, 2020) and tend to like more what they look at (Fig. 2, P2; Bhatnagar & Orquin, 2021; Gluth et al., 2020). Shimojo and co-authors (2003) first proposed this bidirectional effect as a mechanism for the *gaze cascade*, which is the rapidly accumulating attention on the chosen alternative just before (500–750 ms) a choice is expressed. The gaze cascade is a robust phenomenon that has been demonstrated to occur in two-alternative perceptual and preferential decision tasks (Glaholt & Reingold, 2009), multi-attribute choice (Atalay et al., 2012), gambles (Fiedler & Glöckner, 2012), and intertemporal decision making (Fig. 2, P1; Fisher, 2021).

## Perceptual Decision Making: Eye-Movement Models of Search

In *target search,* people search for a predefined object surrounded by other objects on a visual display (Table 1). Search for a specific perfume on the website in Fig. 1 is an example. Target search may occur before people have made a choice, such as when searching for products to consider buying, or after they made a choice, such as when searching for a product they have earlier decided to buy. In *specification search, *people search for information on the attributes of an object (Table 1), such as the price, brand name or color, among information about other objects (Moorthy et al., 1997). We next discuss models of these two types of perceptual decisions (Appendix 2 has modeling details).

### Eye-Movement Models of Target Search

In Hidden Markov Models (HMM), unobserved discrete states that evolve over time according to a Markov Process are assumed to generate the observed eye-movement time series according to some probabilistic model. Over-time variations in the observed eye movements are reflections of these unobserved states and the switches between them. Liechty et al. (2003) were among the first to model latent attention states via HMMs to identify shifts between attention strategies that people use during exploratory search upon advertising exposure.

During search for complex targets, people aim to reduce two types of uncertainties: location and identity uncertainties (Ungerleider & Mishkin, 1982; Appendix 1; Table 1). They do so by switching over time between two attention states in which one of the two subtasks takes precedence (see Liechty et al., 2003). The two hidden states, localization and identification, are assumed to follow a Markov process over time. Van der Lans et al. (2008a; b) developed HMMs to uncover the time path of localization and identification states from eye-movement patterns during target search. The models in question specify the location of each fixation, in pixel coordinates, as a spatial point process. The two attention states are identified via parametric assumptions on that process that are specific to each state. The localization state causes long-amplitude saccades and the identification state short-amplitude saccades (Bullier et al., 1996; Thompson, 2005; Appendix 1). Eye movements in the localization state are driven by the salience of locations in the visual display (Itti & Koch, 2001; Koch & Ullman, 1985; Appendix 1). In the model, salience is defined in terms of pixel-level perceptual features, including colors, brightness, and edges. Computer vision techniques are used to extract these features from digital images of the search display, resulting in (RGB or CIELAB) feature values for each pixel. Because the eye only processes detailed information from a region of about two degrees of visual angle around the point of fixation (Appendix 1), a Normal spatial kernel with a width of two degrees is used to smooth these pixel-level variables. The salience map is then represented as a weighted combination of perceptual features, the weights being represented by individual-level model parameters. These parameters are assumed to follow a Normal distribution and are estimated based on the eye-movement data. They capture the effect of top-down factors on attention (Fig. 2, P5). Although some consider salience to be a purely bottom-up stimulus property (Itti & Koch, 2001), its operationalization as a weighted sum of basic features by Van der Lans et al. (2008a; b) is in line with the idea that salience results from activation or inhibition of visual information that is deemed, respectively, relevant or irrelevant, which is a top-down process (Gaspelin & Luck, 2018; Sawaki & Luck, 2010). Similar probabilistic formulations of salience had been previously proposed (Baddeley & Tatler, 2006; Rutishauser & Koch, 2007). The Van der Lans et al. target search model provides estimates of individual-level salience maps. Further, in the localization state, factors measured at the level of the display (scene organization or display architecture) and the person (task schemas or scanning habits) drive eye movements (Fig. 2, I2, I5). Systematic eye-movement strategies (Spalek & Hammad, 2005) are captured via Markov transition probabilities on the ROIs containing objects to the left and right, or top and bottom, respectively, of the previously fixated ROI. The identification state accommodates the tendency to re-fixate on an object to collect more information on its identity (Smith & Henderson, 2009; Tatler & Vincent, 2009). This model and its extensions (van der Lans et al., 2021) incorporates a range of well-documented neuro-psychological processes (Appendix 1).

Top-down modulation of low-level visual processes involves selectively enhancing visual features that are diagnostic for a search task and suppressing features that are non-diagnostic (Desimone & Duncan, 1995; Fig. 2, P5; Einhäuser, Rutishauser, & Koch, 2008; Rutishauser & Koch, 2007). Bayesian formulations that encapsulate top-down information via a hierarchical prior reflect the mechanism involved (Borji, Sihite, & Itti, 2014; Lee & Mumford, 2003; Torralba et al., 2006). Van der Lans et al. (2008a) manipulate task instructions to enable the decomposition of bottom-up and top-down influences according to such a Bayesian hierarchical structure. Estimation of the model on eye-movement data, collected while participants were searching for brands of laundry detergent on a simulated shopping shelf, revealed that about two thirds of the variation in the salience of products on the shelf was due to bottom-up factors and one third due to top-down factors.

Several components of these search models, such as top-down effects and salience-based and systematic processing, can be used in stand-alone models applied in experiments in which other uncertainties have been strictly controlled for. For example, the relative salience of an object fixated during search can be estimated as the average fixation-weighted Euclidean distance between that object and the target object in the CIELAB color space (van der Lans et al., 2021). Such an operationalization of salience encapsulates the extent to which participants fixate on distractors that are similar to the target, with shared color being a dominant source of visual similarity (Rutishauser & Koch, 2007). Results across a range of products (sunglasses, sneakers, perfumes) revealed that search efficiency gains occur via top-down suppression of perceptual features of competing, *distracto*r products rather than by enhancement of the features of the target product (van der Lans et al., 2021; Fig. 2, P5).

Recent work has captured the time-dependency of eye movements during search and related perceptual decision tasks via first-order autoregressive (AR) model formulations, rather than via HMMs. In AR models, the observed variable (fixations) depends probabilistically on its own past values. AR formulations have been used to capture time-dependencies in eye movements in Generalized Linear Mixed models by Cho, Brown-Schmidt and Lee (2018), and in tree-based Item Response Theory models by Cho, Brown-Schmidt, Boeck and Shen (2020). These models describe the POR (point of regard) of the eyes during search as a sequence of binary micro-decisions. The neuro-psychological support for such formulations, although not used to motivate the original modeling, may come from such phenomena as inhibition of return (IOR; Castel, Pratt, & Craik, 2003), attentional momentum (Pratt, Spalek, & Bradshaw, 1999), and systematic oculomotor tendencies (Spalek & Hammad, 2005; Fig. 2, P5). Looking at time dependencies via a hierarchical Bayes logistic regression, Shi and Trusov (2021) study how people explore search engine (Google) results. They investigate what part of the results page people look at, whether they scroll, which items they look at, and which item they click on. The results show that the prior scan-path affects these micro-decisions, along with top-down effects of search goals and bottom-up effects of the semantic context and the spatial layout of the page.

### Eye-Movement Models of Specification Search

Research in agricultural and food economics has examined specification search for price, nutrition, sustainability and other attributes of products, packaging, and food labels, as reviewed by Van Loo et al. (2018). The purpose of that research is to understand which perceptual features and abstract attributes of objects influence perceptual (“find the most sustainable products on display”) and preferential decisions (“choose the products that fit your preferences best”). Research in this tradition has explored bottom-up and top-down factors, the latter including for example having “no goal” versus having “general health” or more specific “reduce sodium intake” goals (Oswald, Adhikari, & Mohan, 2022). For instance, van Herpen and van Trijp (2011) found that pictorial “traffic light” labels improved search for attributes of food more than textual labels did, even when people were not under time pressure. These studies provide descriptions of specification search as it unfolds in daily life.

Eye movements during specification search have also been studied as part of other than pure specification tasks (see Table 1). For example, research has examined visual exploration of so-called retail feature advertisements, which display multiple (price discounted or otherwise featured) products in a single advertisement. Visual exploration of these ads is a mixed target/specification search task (Table 1), during which people aim to reduce uncertainty about whether a preferred product is present and if so, what its attributes are. Pieters, Wedel and Zhang (2007) examined such a task with a hierarchical Bayes model to capture the multilevel nature of multiple ads nested in such ad displays. They used measures of the visual distinctiveness of the target (perceptual difference between a target ad and its competitors in terms of the sizes of their ROIs -regions of interest) and the heterogeneity of distractors (perceptual differences among the other, competing ads) as predictor variables. Their results showed, in support of fundamental psychological theories (Duncan & Humphreys, 1989), that target distinctiveness facilitated and distractor heterogeneity hampered search. The modelling results enabled optimizing the organization of the ad display to improve search performance for each of the advertised products. Strategy switching during specification search tasks (Liechty et al., 2003) has also been studied, for instance during exploration of theme advertisements (Liechty et al., 2003; Wedel et al., 2008), word-sentence processing (Simola, Salojärvi, & Kojo, 2008), picture viewing (Haji-Abolhassani & Clark, 2013, 2014), and tracking moving targets (Kim, Singh, Thiessen, & Fisher, 2020).

In view of the ubiquity of search and choice behavior by consumers on attribute-by-product displays and the potential costs of errors, the paucity of eye-movement research in this domain is surprising. But, for instance, tracking eye movements on ”balanced score cards” that managers rely on to assess employees (Chen, Jermias, & Panggabean, 2016) revealed factors that support better accounting decisions. Eye-movement research into search and choice on row-column displays has led to qualitatively different insights than obtained by more traditional process tracing methodologies. Research relying on traditional process tracing methodologies, such as information display boards (e.g., Lohse & Johnson, 1996) or one-way mirrors to observe people’s eye movements during decision making (e.g., Russo & Leclerc, 1994) initially identified two or three well-ordered sequential stages in preferential decision making (from screening to evaluation, and then to verification). Likewise, eye-tracking studies initially found that in repeated conjoint choice tasks, participants switch from using attribute-based strategies first to product-based strategies later on (Meißner & Decker, 2010). Research with Hidden Markov Models (HMMs) has amended this orderly, sequential, two or three-stage perspective on search and choice processes. Shi et al. (2013) used a three-layer Hierarchical Hidden Markov Model (HHMM) to represent eye movements made to acquire information on attribute-by-product matrices. Their model has two hierarchically connected layers of unobserved states, where the transitions between states in each layer are governed by a Markov process. Given the states of the first hidden layer, the output layer describes the eye movements using Markov transition probabilities between ROIs that are defined via a row (attribute)-column (product) spatial grid. The first hidden layer consists of two states that represent the latent by-attribute or by-product information acquisition strategies, given the states of the second layer. That second layer contains states that allow for switching between these first-layer strategies. The second-layer states, the authors speculated, might reflect top-down processes that activate a specific information-acquisition strategy. Notably, findings from the HHMM applied to data about choices between laptops presented on a 12 by 4 row-column display are at variance with some of the received knowledge obtained from traditional process tracing methodologies and descriptive modeling. First, rather than starting with attribute-based search, people tend to start and end with product-based information search, while in between relying on attribute-based search. Second, rather than switching only once or twice between attribute-based and product-based search, people switched many times. Third, the switching patterns between attribute- and product-based processing depended on the orientation of the display (attributes-by-products or products-by-attributes).

In another application, Chuk et al. (2020) use a similar two-level HHMM to describe visual information acquisition during choice of one out of two faces. The purpose of the analysis was to identify regions of interest (ROIs) that are unknown in terms of their number, locations, and boundaries, and the saccade patterns between them. The states in the first hidden layer are interpreted as individual-specific ROIs, that is regions on the human face that a participant uses in deciding which face they find more appealing. The second layer of the HHMM captures the transitions between states of the first hidden layer. The fixation locations at the output layer are modelled via a spatial Gaussian distribution. The HHMM was estimated for each individual separately using a (variational) Bayesian approach that determines the optimal number of states automatically. The results reveal two dominant patterns of eye movements. In the first pattern, people fixate on the preferred face sooner, but the accuracy of inferring their final choice from the eye movements is lower. In the second, people transition from exploration to fixating on the preferred face later, but the tendency to fixate on their preferred face is stronger.

## Preferential Decision Making: Eye-Movement Models of Choice

Models of preferential or value-based decision making in marketing and economics traditionally rely on the assumption that people integrate all available information on attributes of the alternatives to arrive at the value or utility of each choice option (Fig. 2, I3). For instance, in sequential search model in economics (Weitzman, 1979), people are assumed to first sort choice alternatives in order of expected marginal utility and then sequentially search the options until the expected utility of (examining) the next alternative is less than the current alternative (Moorthy et al., 1997). During that type of product-based processing, people are assumed to rely on compensatory (Fishbein & Ajzen, 1975; Von Neumann & Morgenstern, 1947) preference models of a linear additive (subjective expected utility) form. Then, the choice outcome corresponds probabilistically to the alternative with maximum utility among the set of all alternatives. Models of such preferential decisions involve the Multinomial Logit or Probit functional forms, which link utility to decision outcomes (Fig. 2, O3). The Mixed Multinomial Logit model is an extension that accommodates unobserved heterogeneity by allowing its parameters to follow a Normal distribution across individuals.

These assumed processes occur during integration tasks (Table 1), but integration does not necessarily involve utility maximization, nor full information, nor linear compensatory choice rules (McFadden, 1974). In real-life decision making, people use various simplifying strategies and choice heuristics to prevent cognitive overload and to balance search benefits and costs (Kahneman, 2011). For instance, choice inertia and habitual choice are common for (frequent) low risk, low-involvement decisions. These phenomena can be readily accommodated in choice models through Markov formulations that include the immediate past decision outcome or via an exponentially smoothed average of past decision outcomes (Guadagni & Little, 1983; Keane, 1997). These formulations in essence capture reinforcement learning where the learning rates are prespecified (equal to one) or estimated (Rescorla & Wagner, 1972).

Reflective, non-habitual, decision making is more prevalent for high-involvement products and contexts. But even there, some information, such as specific attribute-alternative combinations or even entire attributes or alternatives, may not be considered at all, because the expected costs of processing outweigh the expected benefits (Sims, 2003). Thus, to lower cognitive effort, people use simplifying heuristics that are based on only a part of the available information (Payne, Bettman, & Johnson, 1988; 1992). Examples include satisficing (Simon, 1955), lexicographic (Von Neumann & Morgenstern, 1947), and elimination-by-aspects rules (Tversky, 1972). To capture these “boundedly rational” decisions, selection mechanisms have been introduced in choice models to reflect how people narrow down the choice set prior to making a full-information choice. Two-stage nested logit models (McFadden, 1981), joint models of consideration and choice (Roberts & Lattin, 1997), and models that account for decision strategy shifts (Swait & Adamowicz, 2001) are examples. Alternatively, latent binary selection indicators have been included into utility functions as moderators of product attributes, to capture a variety of decision heuristics (Gilbride & Allenby, 2004, 2006; Jedidi & Kohli, 2008). Research incorporating eye-movement data in choice models has revealed that people rely on compensatory (maximizing) decision making in some instances (Glöckner & Herbold, 2011), on satisficing rules that involve people attending to alternatives until a satisfactory one is found in other (low-involvement) instances (Stüttgen et al., 2012), or on hybrid decision strategies that involve a combination of optimal search and satisficing (Reutskaja, Nagel, Camerer, & Rangel, 2011).

To structure the burgeoning field of eye-movement research on decision making, we distinguish three categories of approaches, based on whether eye movements are (1) used as explanatory variables to infer what attributes and choice options (products) are processed (Sect. 4.1), (2) used in psychological models as indicators that moderate evidence accumulation (Sect. 4.2), or (3) modeled endogenously along with choice outcomes based on economic principles (Sect. 4.3) (details of the key models are provided in Appendix 2).

### Preferential Decision Models with Eye-Movements as Explanatory Variables

Eye movements have been used as explanatory variables in choice models to capture the information that people use or ignore in making decisions. *Inattentional blindness* is an automatic phenomenon that occurs when certain objects or attributes of a scene are not noticed (even though they might receive eye fixations) because attention is devoted to another task or object (Mack & Rock, 1998). In the context of multi-attribute choice, attribute inattention (Fig. 2, I3, P5) occurs when certain attributes are not processed (Hensher, Rose, & Greene, 2005), and object inattention occurs when one or more alternatives are not considered in the choice decision (Roberts & Lattin, 1997). But such inattention can be mitigated. When choice tasks in eye-tracking studies are designed to be incentive compatible, participants utilize up to twenty percent more of the attribute information presented to them (Yang, Toubia, & de Jong, 2018). Nonetheless, to accommodate inattention, eye-movement selection indicators (yes-no fixated) have been used as explanatory variables in choice models, as direct effects (Pieters & Warlop, 1999), as moderators of the attributes (part-worths) of products (Balcombe, Fraser, & McSorley, 2015; Yegoryan, Guhl, & Klapper, 2020), or as mediators to explain decision outcomes (Chandon et al., 2009; Pieters & Warlop, 1999).

### Psychological Process Models of Preferential Decision Making

A stream of literature in psychology has developed process models of decision making that are calibrated on response times and choice outcomes. Such Sequential Sampling Models (SSM) (Ratcliff, 1978; Ratcliff, Van Zandt, & McKoon, 1999; Smith & Ratcliff, 2004) assume that evidence (perceptual choice) or utility (preferential choice) on two or more choice alternatives accumulates stochastically, until one alternative has accumulated sufficient evidence to cross a decision threshold. A larger value of the threshold results in more accurate but slower responses. Drift Diffusion Models (DDM) are a special case. In the basic DDM version, the accumulation of evidence on each alternative is described by a Wiener diffusion process (which has independent Gaussian increments). DDMs were originally developed to describe accuracy and latency of two-alternative perceptual decision tasks (Ratcliff, 1978; Smith & Ratcliff, 2004; Voss, Nagler, & Lerche, 2013), but have later also been applied to examine preferential decision making (Dutilh & Rieskamp, 2016; Summerfield & Tsetsos, 2012; Voss, Rothermund, & Voss, 2004).

Commonly, DDMs are fit to preferential decision outcomes assuming an underlying diffusion process according to which a unitary subjective value for each alternative accumulates over time. For example, DDMs have been used to describe choices between pairs of food products using preference ratings for these products provided by participants prior to the choice task as exogenous input (Milosavljevic, Malmaud, Huth, Koch, & Rangel, 2010). DDMs have been fit to choices among multiple alternatives as well, based on the assumption that the differences in accumulated evidence among them favors one of the options and determines choice. Other SSMs describe multi-alternative choice with multiple diffusion processes, each with their own drift rate, which race until the first one crosses a threshold (Usher & McClelland, 2001; Usher, Olami, & McClelland, 2002).

A critical assumption of traditional DDMs is that visual information uptake is randomly distributed across alternatives. Therefore, these models imply that eye movements are uniformly distributed over alternatives (and attributes), which is similar to the implicit assumption in compensatory decision models that people attend equally to all information in the choice task (Glöckner & Herbold, 2011; Orquin et al., 2021). Despite some challenges in generalizing DDMs, and SSMs more generally, to multi-alternative preferential choice in realistic contexts (e.g., Mormann & Russo, 2021), the models provide an important step toward connecting neuro-physiological processes to unobserved preference formation. With a few exceptions (e.g., Satomura, Wedel, & Pieters, 2014), sequential sampling models have yet received limited attention in the marketing and economics literature as process models for decision making. Nonetheless, the Poisson race model, which also rest on sequential sampling of information during stimulus exposure, has been applied to conjoint choice tasks (Ruan, MacEachern, Otter, & Dean, 2008). That model assumes that information signals arrive for each alternative according to a Poisson process (with time-varying rate), until an (integer) threshold is crossed. While that model was fit to only macro-outcomes, choice and latency, Poisson race models for perceptual decisions have used eye-tracking data to express how random chunks of information arrive via eye fixations according to a Poisson process until a threshold is crossed that determines the decision outcome (Wedel & Pieters, 2000).

Extensions of DDMs have included eye movements to describe perceptual (Rangelov & Mattingley, 2020; Tavares, Perona, & Rangel, 2017) and preferential decision-making (Callaway et al., 2021; Cavanagh, Wiecki, Kochar, & Frank, 2014; Towal, Mormann, & Koch, 2013). The key feature of the attentional DDM (aDDM) by Krajbich, Rangel and colleagues (Krajbich et al., 2010; Krajbich, Lu, Camerer, & Rangel, 2012; Krajbich & Rangel, 2011) is that the information on any alternative accumulates only while the eyes fixate on that alternative. The parameters of the model capture the speed of value accumulation, the bias toward the fixated option, and the error variance.

The initial aDDM predicts that alternatives fixated first and fixated more frequently are more likely to be chosen. Still, it does not allow for effects of bottom-up factors (Fig. 2, I1, I2, P3), nor for effects of the accumulating utility on eye movements (Fig. 2, P2; Mormann & Russo, 2021). Thus, it assumes the eye-movement process to be exogenous to the preference formation and choice processes, that is, saccades between choice options are assumed to be random, which is at variance which evidence reviewed here. Recent extensions of the aDDM (Gluth et al., 2020) allow the accumulated value of an option to increase the likelihood that it will be fixated (Fig. 2, P2). This extended model accounts for various eye-movement patterns, including the first fixation and the evolution of fixations on choice alternatives over time. Likewise, Song, Wang, Zhang and Li (2019) have proposed a sequential sampling model in which alternatives with higher (value/utility) uncertainty are more likely to be fixated (Table 1). In their model, eye fixations deliver additional information that reduces the uncertainty about choice alternatives via a Bayesian updating mechanism. It has been shown (Callaway et al., 2021) that with Bayesian updating the selection of which alternative to fixate and when to terminate the process can be formulated as a dynamic optimization problem, in which (costly) eye movements are allocated optimally over time to acquire information that reduces uncertainty about the values of the alternatives.

### Economic Models of Preferential Decision Making

Recent models of preferential decision-making aim to predict future choice between multiple alternatives while endogenizing the complete eye-movement process. For example, Martinovici et al. (2021) use not just the final fixations, as in research on the gaze cascade (Shimojo et al., 2003), or the sum of fixations as in aDDMs, but the entire trajectory of eye movements for each of the choice alternatives to predict preferential decision making in a naturalistic choice task. They develop a joint model of eye movements and choice outcomes that describes the gaze on product ROIs over time as a set of covarying higher-order polynomials. It enables prediction of the final choice from gaze patterns well before the actual choices are implemented, out-of-sample. It is a predictive model, however, that is agnostic about the underlying interplay between attention and preference formation prior to choice.

Researchers have recently applied economic modeling frameworks (Gabaix, Laibson, Moloche, & Weinberg, 2006; Moorthy et al., 1997; Weitzman, 1979) to eye movements, in order to describe information search and choice for high-involvement products (e.g., laptops, cell phones). These frameworks describe information search as a sequential process with an optimal solution. At each step in the process, a decision maker examines the alternative with the highest utility and stops when the realized utility of that alternative is larger than the expected utility of the next alternative (Ursu et al., 2021; Yang et al., 2015). Information search in these models is thus determined by the expected value that the next piece of information provides. The models in question simultaneously embed micro- (fixation) and macro- (choice) decisions in a utility maximization, multinomial logit, modeling framework. The models assume that people use Bayesian updating of their prior uncertainty about the value of an attribute level (Table 1) with a noisy chunk of information obtained by fixating a product attribute, resulting in an updated product value. The expected utility is modeled as a weighted sum of the part-utilities of the attribute levels. The utility of search (and thus implicitly its cost) is specified as a function of eye-movement characteristics, such as the saccade length and direction (Ursu et al., 2021; Yang et al., 2015) to allow for local clusters of fixations and for horizontal and vertical saccade tendencies. Unobserved heterogeneity in the models’ parameters across individuals is accommodated via a Normal distribution. The results of that research show that integrating eye movements into models of search and choice improves parameter estimates, the understanding of the choice process, as well as out-of-sample predictions.

The appeal of such economic approaches is that they (1) integrate choice and eye-movement decisions in a single framework, (2) postulate a plausible economic (cost-benefit) mechanism for eye-movement decisions (Callaway et al., 2021), and (3) assume that utility/value evolves during the decision process and affects fixations (Gluth et al., 2020; Ursu et al., 2021). However, a limitation of current implementations is that known influences of visual factors and attentional processes (Fig. 2, bottom up: I1, I2, P3, and top down: I4, P4, P5) are not accommodated or only in a basic way. Also, the assumed (one-step) forward-looking mechanism remains as yet unverified.

Research for low-involvement value-based decisions (e.g., snacks) has also relied on an utility framework, but has specified different underlying processes (Reutskaja et al., 2011; Stüttgen et al., 2012). For low-involvement decisions, target search is more likely a main component of the choice task (Table 1), with people relying on the perceptual salience of objects, habitual decision making, and satisficing choice rules. For instance, in modeling choice of one out of a large set of products under time pressure, it was assumed that people may stop and choose after having fixated all alternatives at least once or after running out of time (Reutskaja et al., 2011). This assumption is in line with a satisficing choice heuristic where search stops after the value of the currently fixated alternative exceeds a reservation value.

Stüttgen et al. (2012) extended the target search model of van der Lans et al. (2008a) by including a third state in the HMM, in addition to the localization and identification states. The model specifies a satisficing rather than maximizing process (Simon, 1955). Specifically, in the third, termination state an individual chooses a product that is satisfactory, as follows (products that are undetermined are chosen with a probability close to zero). The individual assigns the status satisfactory, unsatisfactory, or undetermined to each product at each fixation. If a product (here, instant noodles) has not been fixated, its status is undetermined. To determine whether a product is satisfactory, the individual needs to fixate all attributes of that product and the value of each attribute needs to be acceptable. The model accounts for strategy switching, where the switching probabilities depend on the status of the products and the number of prior fixations. It allows people to proceed to a new choice option before returning and making a final choice, however, which is at variance with a pure satisficing heuristic (Gilbride & Allenby, 2004, 2006; Jedidi & Kohli, 2008) or the process assumed in aDDMs (Gluth et al., 2020; Krajbich et al., 2010; Song et al., 2019).

## Conclusions and Opportunities for Future Research

Eye movements offer a unique window on perceptual, cognitive, and evaluative processes of people who are engaged in decision making tasks. They afford tests of fundamental theories, new insights into search and choice phenomena, and predictions of future decisions. Understanding how complex decisions are made in real life requires recognizing that real-life decision tasks are mixtures of elementary tasks through which people try to reduce multiple uncertainties (Table 1). Therefore, a key component of our framework in Table 1 and Fig. 2 is the idea that people switch between strategies to reduce the multiple uncertainties inherent to complex decision tasks. In addition, the execution of these tasks is subject to a myriad of bottom-up and top-down factors that influence the perceptual, cognitive, and evaluative processes that drive observable micro- (eye movements) and macro- (choice) decision outcomes (Fig. 1). Psychometric, econometric, and statistical models of these micro- and macro-decisions have been developed for somewhat different decision-making contexts and are therefore based on different assumptions tailored to these contexts. To advance insights into search and choice, a synthesis of these models is called for. The present review aims to provide a first step toward such a synthesis, and the research agenda that we propose next serves that purpose.

First, while existing eye-movement models of target (van der Lans et al., 2008a; b; 2021) and specification search (Shi et al., 2013) capture perceptual decision tasks (Table 1) relatively accurately, there are opportunities to improve upon them. Extant models of target and specification search are disconnected and cannot directly be used to predict choice outcomes, which presents an opportunity for future research. In addition, these models have relied on a priori specified perceptual features and regions of interest (ROIs). Further research can establish whether such prespecified variables most accurately capture the visual inputs that people use to process information during perceptual decision tasks. Advances in computer vision and machine learning could be used to (automatically) extract a large variety of characteristics of images, video, and text. Furthermore, eye-movement patterns themselves can be used to extract ROIs that are relevant for the decision task at hand (Chuk et al., 2020). Those data could be used in learning models (Ursu et al., 2021; Yang et al., 2015) to better understand what information people use during identification and specification processes (see Table 1). Further, the rich body of knowledge on eye movements during reading (Rayner, 1998; Rayner, Schotter, Masson, Potter, & Treiman, 2016) may inform future studies that aim to refine models of people’s information acquisition from text during decision making.

Second, there are opportunities to extend DDMs and, similarly, SSMs. These models have been used to fit preferential decisions by postulating mechanisms of how the accumulation of evidence reduces decision uncertainty, similar to those for perceptual decision making (Callaway et al., 2021; Dutilh & Rieskamp, 2016). Several directions for improved theorizing and modeling remain: (1) extant research often infers the plausibility of the underlying sequential sampling processes from the statistical fit between inputs and outputs, rather than by examining the processes directly through eye movements. (2) DDMs typically rely on the assumption that visual information uptake is uniformly distributed across alternatives and attributes (for an exception see Gluth et al., 2020). This assumption is at variance with evidence that attention tends to be directed to higher valued options or the emerging winner during choice tasks (Martinovici et al., 2021; Meißner, Musalem, & Huber, 2016; Pieters & Warlop, 1999; Shimojo et al., 2003). (3) Preferences are often assumed to be fixed and are measured prior to the decision task. The drift in a DDM is then to be interpreted as the accumulation of information on those preferences retrieved from memory (Milosavljevic et al., 2010). While this simplification may provide a reasonable description of habitual, perceptual decision making, it hampers the successful application of DDMs to high-involvement, preferential choice contexts in which perceived utility of choice options and eye movements toward them may interact. The attentional DDM takes a step forward by incorporating eye movements into the value-accumulation process (Krajbich et al., 2010), but the mechanism driving eye movements is still mostly assumed exogenous (Mormann & Russo, 2021). In addition, the assumption that all attention reflects or adds positive utility requires tests in contexts where people attend to negative outcomes to avoid. More research is thus needed on the dynamic interplay of attention and value accumulation in DDMs (Fig. 2, P1 and P2), also because that interplay causes violations of IIA decision making (Gluth et al., 2020). IIA (Independence of Irrelevant Alternatives) specifies, restrictively, that the ratio of choice probabilities between two alternatives should not depend on the presence of a third alternative (Louie, Khaw, & Glimcher, 2013; Luce, 1977). Recent work that extends the aDDM by formulating the probability that an alternative is fixated as a (logistic) function of its accumulated value (Gluth et al., 2020) holds promise. Further, research is needed to alleviate the assumption in extant DDMs that decisions are based on accumulation of a unitary subjective value, by allowing for representations of multi-attribute decision making similar to those in economic models of choice. Some DDMs for perceptual decisions already incorporate such formulations. For example, Noguchi and Stewart (2018) propose an evidence accumulation model based on observations from process-tracing and eye-movement models in which decisions are based on the accumulation of pairwise comparisons of attributes.

Third, econometric models of search and choice assume that people are forward looking and make a tradeoff between choosing now based on the available information or using eye movements to search for more information and then making a decision (Ursu et al., 2021; Yang et al., 2015). The key assumption that people are (one-step ahead) forward looking when making mostly subconscious eye movements remains to be verified, however. That assumption might hold for high-involvement contexts involving specification search, but less so in low-involvement contexts where target search dominates preferential choice. A further limitation of current econometric applications is that several key attention processes (Fig. 2, P3 and P5) are not considered or are included via fairly restrictive assumptions. For example, visual factors, which have a large impact on choice decisions (Orquin et al., 2021), are typically absent. Extant models do not account for consecutive refixations on the same object (Chandon et al., 2009; Krajbich et al., 2010; Martinovici et al., 2021; Yang et al., 2015), which is a striking characteristic of scan-paths across a wide range of tasks (Liechty et al., 2003; Musalem, Montoya, Meißner, & Huber, 2021; Rayner, 1998; van der Lans et al., 2008b).

Fourth, despite the different approaches taken in the models developed in psychology, marketing and economics, there are also some remarkable similarities. As a case in point, econometric (random utility) choice models and some SSMs used in psychology are mathematically equivalent (Webb, 2019). Further, the threshold-crossing phenomena in SSMs resemble satisficing (Glimcher, 2010; Reutskaja et al., 2011; Stüttgen et al., 2012). DDMs are mathematically equivalent to Bayesian decision models (Bitzer, Park, Blankenburg, & Kiebel, 2014). The Bayesian updating mechanism by which fixations reduce uncertainty in process models in psychology (Callaway et al., 2021; Song et al., 2019) is almost identical to the mechanism that has been assumed in models of search and choice in marketing and economics (Ursu et al., 2021; Yang et al., 2015). Finally, the hierarchical Bayesian priors that have been used in psychology and computer vision to capture top-down influences on eye-movements (Borji et al., 2014; Torralba et al., 2006) are identical to those implemented in statistical models of search in marketing (van der Lans et al., 2008a). Such similarities in approaches developed in rather independent streams of research not only reveal convergent validity of assumptions underlying different models, but also provide touchpoints to further integrate models from psychology, economics, and marketing. This review has tried to lay a foundation for such an integration by incorporating the elementary tasks and cognitive processes involved in perceptual and preferential decision making in a single conceptual framework (Table 1; Fig. 2).

Fifth, more theoretical and empirical work on the role of tasks and task switching in search and choice is called for. We have noted that decisions in real-life contexts comprise of elementary subtasks (Table 1) that may, for example, involve both target search and specification search, or habitual and reflective decision making. Future research could integrate mathematical representations of these processes by capitalizing on representations of task/strategy switching and existing communalities between model formulations. Strategy switching has already been demonstrated for micro-decisions in a wide range of tasks (Chuk et al., 2020; Shi et al., 2013; Stüttgen et al., 2012; van der Lans et al., 2008a; b; Wedel et al., 2008). Whereas dual-process theories for macro-decisions abound (Damasio, 1994; Glimcher, 2010; Kahneman, 2011), and initial evidence for extensive strategy switching during these decisions comes from eye movement recordings (Venkatraman et al., 2014), more work is awaited that incorporates strategy switching in psychological (SSM) and econometric (sequential search) models and applies these to more fully understand how people switch between decision strategies, for example based on cost-benefit tradeoffs (Payne et al., 1988; Venkatraman et al., 2014).

In closing, the study of search and choice in natural conditions requires strong theory as well as sound psychometric, econometric, and statistical models to infer the underlying perceptual, cognitive and evaluative processes. Marketing and economics have provided a fruitful testing ground for theories and models of attention and decision making, because of their focus on consumer decision making toward complex pictorial and textual, static, dynamic, and multisensory stimuli in natural contexts. Eye-movement data have come to play a critical role. Naturally, therefore, research, product and service companies have embraced eye tracking as a tool. With eye tracking via regular webcams being a low-cost option, eye-movement recording is already implemented in smart phones, car navigation, and head-mounted virtual reality devices. Measures produced as a by-product of eye movement recording, such as fixation durations, micro-saccades, distance between eye and stimulus, pupil dilations, and facial expressions of emotions, could be further exploited (Pieters & Wedel, 2020). Given those developments, understanding, predicting, and improving decision making in practice would benefit greatly from adopting the models reviewed here, and the extensions and generalizations that we hope the proposed framework stimulates.

## Appendix 1: Neural Foundations of Eye Movements During Search and Choice

Light is projected through the cornea and the lens of the eye, on the retina. The photoreceptors in the retina process color (cones; in the fovea), luminance (rods; in the periphery) and contrast (bipolar and ganglion cells), amongst others. Via the fovea, the eye extracts high-acuity information from a region of about 2 degrees of visual angle (Palmer, 1999). Signals from the retina are sent via the optic nerve to the Lateral Geniculate Nuclei (LGN; in the Thalamus). From there on information of the two eyes is combined and passed (via the optic radiation) to area V1 in the Occipital Lobe. The visual areas in this lobe form a hierarchy and specialize in the processing of *low-level visual features*, such as edges (V1, V2), depth (V2), contours (V2), simple shapes (V4), colors (V4), and self-(V5), and object (V6) motion. These areas are organized as retinotopic maps.

The basic perceptual features that are processed *bottom-up*, pre-attentively via areas V1-V4 (Treisman & Gelade, 1980; Wolfe, 1994) are combined to form a *salience map* (Itti & Koch, 2001; Itti et al., 1998; Koch & Ullman, 1985). The salience map is a retinotopic map that represents the conspicuity of locations in the visual field. It is represented in V1, the Superior Colliculus (SC) and the Frontal Eye Fields (FEF) (Koch & Ullman, 1985; Niebur & Koch, 1998; Treue, 2003), where its representation may be short-lived (Donk & Soesman, 2010).

These areas V1-V6 output information via two streams, the ventral (“what”) and the dorsal (“where”) stream (Ungerleider & Mishkin, 1982). The *ventral stream* processes mostly foveal information, connects to the Inferotemporal Cortex (ITC) and is concerned with object recognition (Bar, 2004). The ITC projects into the Perirhinal Cortex and Hippocampus amongst others, which are involved in memory, and into the Prefrontal Cortex (PFC). The PFC is involved in planning and reflective *goal-directed decisions*, while the (dorsolateral) striatum is involved in *habitual or reflexive decisions; *both regions are thought to encode value (Daw & O’Doherty, 2014; Glimcher, 2010).

The *top-down influence* of *goals*, for example, originates in the PFC (Desimone & Duncan, 1995), while *value-driven attentional capture*, whereby stimuli associated with a reward attract attention involuntarily, involves dopamine production (which confers motivational salience) from the basal ganglia (Berridge, 2012; Hayhoe & Ballard, 2005). Top-down influences occur along the entire visual hierarchy in areas V1-V5, ITC, SC, and PPC (Corbetta & Shulman, 2002; Tipper, Weaver, & Houghton, 1994) and enhance task-relevant and suppress task-irrelevant features and objects (Sawaki & Luck, 2010).

The *dorsal stream* processes spatial information mostly from the peripheral retina and is connected to the Posterior Parietal Cortex (PPC) which is involved in the location of and planning of action toward objects and in integrating sensory information. The Retrosplenial Cortex (RSC) holds a representation of scenes based on coarse global information and activates scene schemas in the Parahippocampal Cortex (PHC; Bar, 2004). The PHC primes specific object representations in the ITC (Bar, 2004) and is thereby responsible for contextual guidance of eye movements.

The SC and FEF are involved in the production of *eye movements*. The SC receives input directly from the LGN and directs the eyes to salient locations. The FEF direct eye movements to remembered stimuli and are involved in both the facilitation of voluntary saccades and in the inhibition of reflexive saccades that originate in the SC (Rafal, Machado, Ro, & Ingle, 2000). Specific areas of the SC and FEF that are part of the dorsal stream are involved in long-amplitude saccades, whereas other areas of the SC and FEF that are part of the ventral stream produce short-amplitude saccades (Bullier et al., 1996; Thompson, 2005). The SC is also involved in the *Inhibition of Return* (IOR; Sapir, Soroker, Berger, & Henik, 1999), which temporarily inhibits the return of the eyes to previously attended locations or objects (Castel et al., 2003).

## Appendix 2: Details of Selected Search and Choice Models

This appendix details some of the models described in the main text, using the following notation. At time t, a visual scene is comprised of coordinates $\left( l_{1},l_{2} \right)$, with $l_{1}=1,..,L_{1}$ and $l_{2}=1,..,L_{2}\mathrm {.}$ Image content is given by $\mathrm {M}_{\mathrm {kt}}\left( \mathrm {l}_{\mathrm {1,}}, \mathrm {l}_{\mathrm {2}} \right)$, $\mathrm {k=1,\ldots ,K}$, for K visual features, and $\mathrm {h\, =\, 1,\ldots ,H}$ ROIs with $\mathrm {D}_{ht}\left( l_{1},l_{2} \right) =1$ if location $\left( l_{1},l_{2} \right)$ falls into ROI h at time t, and zero otherwise. If the images are stationary, the subscript t is dropped. The vector $\mathrm {x}_{\mathrm {i,t}}=\left( l_{1},l_{2} \right)$ represents the location of fixation t for individual i. The entire scan-path is denoted as $\mathrm {x}_{\mathrm {i,1:T}}$.

### Model of Target Search

Using a spatial point process, van der Lans et al. (2008b) model the location of each fixation t for person i in image-pixel coordinates $\left( l_{1},l_{2} \right) {.\, }$ The explanatory variables $\mathrm {V}_{\mathrm {itj}}\left( \mathrm {l}_{\mathrm {1,}}, \mathrm {l}_{\mathrm {2}} \right)$, $\mathrm {j=1,...,J}$ are features $\mathrm {M}_{\mathrm {k}}\left( \mathrm {l}_{\mathrm {1,}}, \mathrm {l}_{\mathrm {2}} \right)$ and ROIs of packages or text of $\mathrm {m\, =\, 1,..,M}$ brands, with $\mathrm {D}_{m}^{pack}\left( l_{1},l_{2} \right) =1$ and $\mathrm {D}_{m}^{text}\left( l_{1},l_{2} \right) =1$ if location $\left( l_{1},l_{2} \right)$ falls into the package or text. The individual-specific effects ${\uptheta }_{\mathrm {i,j\, }}$ of each explanatory variable $\mathrm {V}_{\mathrm {itj}}\left( \mathrm {l}_{\mathrm {1,}}, \mathrm {l}_{\mathrm {2}} \right)$ depend on whether that person is in the localization or in the identification state. The HMM likelihood of the entire scan-path $\mathrm {x}_{\mathrm {i,1:T}}$ of individual $\mathrm {i}$ is:

A.1 $$\begin{aligned}&\mathrm {p}\left( \mathrm {x}_{\mathrm {i,1:T}}\vert {\Theta }_{\mathrm {i\, }} \right) \mathrm {=}\prod \limits _{s=1}^2 {\mathrm {N}\left( {\uptheta }_{\mathrm {i,s\, }}{|}\mu _{s},{\Sigma }_{\mathrm {s}} \right) } \left\{ \sum \limits _{\mathrm {s}_{\mathrm {2}}\mathrm {=1}}^\mathrm {2} {{\ldots }\sum \limits _{\mathrm {s}_{\mathrm {t}_{\mathrm {i}}}\mathrm {=1}}^\mathrm {2} \prod \limits _{\mathrm {t=2}}^{\mathrm {T}_{\mathrm {i}}} {\uppi }_{\mathrm {s}_{\mathrm {t}}\vert \mathrm {s}_{\mathrm {t-1}}} } \mathrm {p}_{s}\left( \mathrm {x}_{\mathrm {i,t}}\mathrm {=}\left( \mathrm {l}_{\mathrm {1,}}, \mathrm {l}_{\mathrm {2}} \right) {|}{\uptheta }_{\mathrm {i,s}},\mathrm {V}_{\mathrm {it,s}}\left( \mathrm {l}_{\mathrm {1,}}, \mathrm {l}_{\mathrm {2}} \right) \right) \right\} ,\nonumber \\ \end{aligned}$$

where ${\upmu }_{\mathrm {s}}$ and ${\Sigma }_{\mathrm {s}}$ are the mean and covariance matrix of the multivariate Normal heterogeneity distribution of ${\uptheta }_{\mathrm {i,s\, }}$. The Markov transition probabilities between the localization and identification states are: ${\uppi }_{s_{t}{|}s_{t-1}}$, with $s_{t}\in \left\{ 1,2 \right\} ;\, {\uppi }_{\mathrm {s}_{\mathrm {2}}{|}\mathrm {s}_{1}}={\uppi }_{\mathrm {1}}$ represent the initial state probabilities. The vector of explanatory variables $\mathrm {V}_{\mathrm {it,s}}\left( \mathrm {l}_{\mathrm {1,}}, \mathrm {l}_{\mathrm {2}} \right)$ for the localization state (s$=$1) are, first, pixel-level features $\mathrm {M}_{k}\left( \mathrm {l}_{\mathrm {1,}}, \mathrm {l}_{\mathrm {2}} \right)$. Second, systematic strategies are captured via $\mathrm {D}_{{\mathrm {left-right(}x}_{i,t-1})}^{pack}\left( \mathrm {l}_{\mathrm {1,}}, \mathrm {l}_{\mathrm {2}} \right) \,$ and $\mathrm {D}_{{\mathrm {top-bottom(}x}_{i,t-1})}^{pack}\left( \mathrm {l}_{\mathrm {1,}}, \mathrm {l}_{\mathrm {2}} \right) \,$ which represent the ROIs to the left and right, or top and bottom, respectively, of the previously fixated package. Third, in the identification state (s$=$2) the tendency to re-fixate on a text or package ROI is captured via $\mathrm {D}_{{\mathrm {refix(}x}_{i,t-1})}^{pack}\left( \mathrm {l}_{\mathrm {1,}}, \mathrm {l}_{\mathrm {2}} \right) \,$ and $\mathrm {D}_{{\mathrm {refix(}x}_{i,t-1})}^{text}\left( \mathrm {l}_{\mathrm {1,}}, \mathrm {l}_{\mathrm {2}} \right)$. The probability of fixating on a location is:

A.2 $$\begin{aligned} \mathrm {p}_{s}\left( \mathrm {x}_{\mathrm {i,t}}\mathrm {=}\left( \mathrm {l}_{\mathrm {1,}}, \mathrm {l}_{\mathrm {2}} \right) {|}{\uptheta }_{\mathrm {i,s\, }},\mathrm {V}_{\mathrm {it,s}}\left( \mathrm {l}_{\mathrm {1,}}, \mathrm {l}_{\mathrm {2}} \right) \right) \mathrm {=}\frac{{\mathrm {a}_{it,s}\left( \mathrm {l}_{\mathrm {1}}, \mathrm {l}_{\mathrm {2}} \right) }^{\mathrm {2}}}{\sum \limits _{\mathrm {l}_{\mathrm {1}}{\mathrm {,l}}_{\mathrm {2}}} {\mathrm {a}_{it,s}\left( \mathrm {l}_{\mathrm {1}}, \mathrm {l}_{\mathrm {2}} \right) }^{\mathrm {2}} }, \end{aligned}$$

with $\mathrm {a}_{it,s}\left( \mathrm {l}_{\mathrm {1}}, \mathrm {l}_{\mathrm {2}} \right) ={\mathrm {V}_{\mathrm {it,s}}\left( \mathrm {l}_{\mathrm {1,}}, \mathrm {l}_{\mathrm {2}} \right) }^{'}{\uptheta }_{\mathrm {i,s\, }}$. Here, $\mathrm {V}_{\mathrm {it,1}}\left( \mathrm {l}_{\mathrm {1,}}, \mathrm {l}_{\mathrm {2}} \right)$ contains all explanatory variables and $\mathrm {V}_{\mathrm {it,2}}\left( \mathrm {l}_{\mathrm {1,}}, \mathrm {l}_{\mathrm {2}} \right)$ the variables that affect identification. van der Lans et al. (2008a) extend the model by including top-down influences on salience, via a between-subjects experiment with $\mathrm {g(i)\, =\, 1,\ldots ,G}$ search goals of participant i as experimental conditions. The individual-level parameters ${\uptheta }_{\mathrm {i,1:K\, }}$ can then be decomposed into bottom-up (${\upmu )}$ and top-down $\mathrm {(}{\upmu }_{\mathrm {g(i)}})$ influences, according to a hierarchical structure, ${\uptheta }_{\mathrm {i,1:K,\, s=1\, }}{\sim N}\left( {\upmu +}{\upmu }_{\mathrm {g(i)}}, {\Sigma }_{\mathrm {s=1}} \right)$, with Normal priors on ${\upmu }$ and ${\upmu }_{\mathrm {g(i)}}$ and a Wishart prior on ${\Sigma }_{\mathrm {s=1}}$.

### Model of Specification Search

Shi et al. (2013) propose a three-layer HHMM to analyze specification search, which involves three sets of Markovian transition probabilities:

1. The transition probabilities between higher order states, $\mathrm {R}_{\mathrm {t}}$, of the upper layer, ${\uppi }_{\mathrm {R}_{\mathrm {t}}{|}\mathrm {R}_{\mathrm {t-1}}}$
2. The transition probabilities between the two states, $\mathrm {S}_{\mathrm {t}}$, of the middle layer that represent by-attribute and by-product processing, given the states of the upper layer, ${\uppi }_{\mathrm {S}_{\mathrm {t}}{|}\mathrm {S}_{\mathrm {t-1}}}^{\mathrm {R}_{t}}$.
3. The probabilities of eye movements between cells on the display given the states of the middle layer $\mathrm {P}^{\mathrm {S}_{\mathrm {t}}}\left( \mathrm {x}_{\mathrm {i,t}}{|}\mathrm {x}_{\mathrm {i,t-1}} \right)$. Here, $\mathrm {P}^{1}\left( \mathrm {x}_{\mathrm {i,t}}{|}\mathrm {x}_{\mathrm {i,t-1}} \right)$ is a function of $\mathrm {D}_{\mathrm {attr}\left( \mathrm {x}_{\mathrm {i,t-1}} \right) }\left( \mathrm {x}_{\mathrm {i,t}} \right) ,$ attribute ROIs, capturing a by-attribute tendency and $\mathrm {P}^{2}\left( \mathrm {x}_{\mathrm {i,t}}{|}\mathrm {x}_{\mathrm {i,t-1}} \right)$ a function of $\mathrm {D}_{\mathrm {prod}\left( \mathrm {x}_{\mathrm {i,t-1}} \right) }\left( \mathrm {x}_{\mathrm {i,t}} \right) \,$, product ROIs capturing a by-product tendency.

The full HHMM model likelihood is:

A.3 $$\begin{aligned}&\mathrm {p}\left( \mathrm {x}_{\mathrm {i}}{|\pi } \right) \nonumber \\&\quad \mathrm {=}\sum \limits _{\mathrm {R}_{\mathrm {2}}=1}^3 {{\ldots }\sum \limits _{\mathrm {R}_{\mathrm {T}}=1}^3 \left( \sum \limits _{\mathrm {S}_{\mathrm {2}}=1}^2 {{\ldots }\sum \limits _{\mathrm {S}_{\mathrm {T}}=1}^2 {{{\uppi }_{\mathrm {R}_{\mathrm {1}}}{\uppi }_{\mathrm {S}_{1}}^{\mathrm {R}_{1}}\, \mathrm {P}}^{\mathrm {S}_{1}}\left( \mathrm {x}_{\mathrm {i,1}} \right) \prod \limits _{\mathrm {t=2}}^{\mathrm {T}_{\mathrm {i}}} {{\uppi }_{\mathrm {R}_{\mathrm {t}}{|}\mathrm {R}_{\mathrm {t-1}}}{\uppi }_{\mathrm {S}_{\mathrm {t}}{|}\mathrm {S}_{\mathrm {t-1}}}^{\mathrm {R}_{t}}} } } \mathrm {P}^{\mathrm {S}_{\mathrm {t}}}\left( \mathrm {x}_{\mathrm {i,t}}{|}\mathrm {x}_{\mathrm {i,t-1}} \right) \right) .}\nonumber \\ \end{aligned}$$

### DDM of Preferential Decision Making

The attentional DDM (aDDM) (Krajbich et al., 2010; Krajbich et al., 2012; Krajbich & Rangel, 2011) specifies that the rate of accumulation (drift) of the decision value depends on the product or price ROI that the eyes fixate on. Alternative j is selected if its accumulated value $\mathrm {v}_{ijt}$ is the first to cross some threshold $\tau _{i}$: $\mathrm {Y}_{\mathrm {i}}\mathrm {=j,\, if} \quad \left| \mathrm {v}_{ijt} \right| >\tau _{i}$, with $\left| \mathrm {v}_{ikt} \right| \le \tau _{i}\, \forall k=1,..,J\cap k\ne j$. A larger value of the threshold $\tau _{i}$ result in more accurate but slower responses. For a product fixation, the drift is:

A.4 $$\begin{aligned} \mathrm {dv=\phi (}\mathrm {z}_{1}\mathrm {-\beta }\mathrm {z}_{2}\mathrm {)dt+\upsilon dW}, \end{aligned}$$

for a price fixation the drift is $-{\upphi }\left( \mathrm {z}_{2}\mathrm {-\beta }\mathrm {z}_{1} \right)$. Here, $\mathrm {z}_{1}$ and $\mathrm {z}_{2}$ are exogenous measures of product preference and price. $\mathrm {dW}$ is Gaussian noise. The parameters of the model are the drift ${\upphi }$ (for the second alternative the drift is -${\upphi )}$, the bias toward the fixated option ${\upbeta }\in (0,1)$, and error standard deviation ${\upupsilon }$.

### Economic Models of Search and Choice

The model by Yang et al. (2015) is based on the assumption that consumer’s uncertainty about the true value of an attribute level $\mathrm {l}$ of a product, $\mathrm {Z}_{\mathrm {j,m}}$ (with $\mathrm {l}= 1,.., {\mathrm {L}_{\mathrm {m}}})$ is reduced by extracting an (unknown) amount ${\upeta }$ of unbiased information through an eye fixation. Assuming a uniform distribution of prior beliefs across attribute levels, the posterior probabilities that attribute m of alternative j equals $\mathrm {l}$ (with $\mathrm {l\, }= 1,.., {\mathrm {L}_{\mathrm {m}}})$, given that person i has made $\mathrm {N}_{\mathrm {ijm}}$ fixations on it, are:

A.5 $$\begin{aligned} \mathrm {p}\left( \mathrm {Z}_{\mathrm {j,m}}\mathrm {=l} \right) \mathrm {=}\left\{ {\begin{array}{*{20}l} \frac{\mathrm {exp}\left( {{\upeta \cdot N}}_{\mathrm {ijm}} \right) }{{\mathrm {L}_{\mathrm {m}}}\mathrm {-1+exp}\left( {{\upeta \cdot N}}_{\mathrm {ijm}} \right) } &{}\quad \mathrm {if\, l\, is\, the\, true\, level\, of\, attribute\, m,}\\ \frac{\mathrm {1}}{{\mathrm {L}_{\mathrm {m}}}\mathrm {-1+exp}\left( {{\upeta \cdot N}}_{\mathrm {ijm}} \right) } &{} \quad \mathrm {otherwise.\, }\\ \end{array} } \right. \end{aligned}$$

The expected choice utility, $\mathrm {U}_{\mathrm {ij(c)}},$ which is accrued if product j is chosen, is modeled as a probability weighted sum of the part-utilities (${\upmu }_{\mathrm {i,m,l}})$ of the attribute levels:

A.6 $$\begin{aligned} \mathrm {U}_{\mathrm {ij(c)}}=\sum \limits _{\mathrm {m=1}}^\mathrm {M} \sum \limits _{\mathrm {l=1}}^{{\mathrm {L}_{\mathrm {m}}}} {\mathrm {p}\left( \mathrm {Z}_{\mathrm {j,m}}\mathrm {=l} \right) {\upmu }_{\mathrm {i,m,l}}}. \end{aligned}$$

The utility of search, $\mathrm {U}_{\mathrm {i(s)}}$, is specified as a linear function of the saccade length and of horizontal and vertical tendencies. Consumers face the following maximization problem:

A.7 $$\begin{aligned} {\mathop {\text{ max }}\limits _{\mathrm {q}}} \left\{ {\mathop {\text{ max }}\limits _{\mathrm {q=j}}}{\mathrm {U}_{\mathrm {ij(c)}}\left( N_{\mathrm {ij}}\mathrm {,\eta ,}{\upmu }_{\mathrm {i,m}} \right) ,} \,\,{\mathop {\text{ max }}\limits _{\mathrm {m}}} \left( \mathrm {U}_{\mathrm {i(s)}}\mathrm {+}\sum \limits _{\mathrm {l=1}}^{{\mathrm {L}_{\mathrm {m}}}} {\mathrm {p}\left( \mathrm {Z}_{\mathrm {j,m}}\mathrm {=l} \right) } \,{\mathop {\text{ max }}\limits _{\mathrm {j}}}\, {\mathrm {U}_{\mathrm {ij(c)}}\left( \mathrm {N}_{\mathrm {ij}}^{{*}}\mathrm {,\eta ,}{\upmu }_{\mathrm {i,m}} \right) } \right) \right\} .\nonumber \\ \end{aligned}$$

Equation (A.7) specifies a person i’s next action q as a choice between a) stopping search and choosing option j, which results in choice utility ${\mathop {\text{ max }}\limits _{\mathrm {q=j}}} {\mathrm {U}_{\mathrm {ij(c)}}\left( \mathrm {N}_{\mathrm {ij}}\mathrm {,\eta ,}{\upmu }_{\mathrm {i,m}} \right) }$, and b) continuing search and fixating the next attribute m of alternative j, which maximizes search utility $\mathrm {U}_{\mathrm {i}\left( \mathrm {s} \right) }$ plus the expected maximum utility if the individual would stop after this next fixation, which equals $\sum \nolimits _{\mathrm {l=1}}^{\mathrm {L}_{\mathrm {m}}} {\mathrm {p}\left( \mathrm {Z}_{\mathrm {j,m}}\mathrm {=l} \right) } {\mathop {\text{ max }}\limits _{\mathrm {j}}} {\mathrm {U}_{\mathrm {ij(c)}}\left( \mathrm {N}_{\mathrm {ij}}^{{*}}\mathrm {,\eta ,}{\upmu }_{\mathrm {I,m}} \right) }$, where $\mathrm {N}_{\mathrm {ij}}^{{*}}$ is the vector with the number of fixations on option j.

Zhang, Ursu and Erdem (2020) assume consumer i’s prior belief about brand j’s value is $\mathrm {N}\left( {\upalpha }_{\mathrm {ij,0}}, {\upsigma }_{\mathrm {ij,0}}^{\mathrm {2}} \right)$. The prior is updated with a noisy signal, ${\upeta }_{\mathrm {ijm,t}}{\sim N}\left( {\upmu }_{\mathrm {ijm}}, {\upomega }_{\mathrm {ijm}}^{\mathrm {2}} \right)$ from an eye fixation on attribute m and brand j at time t, resulting in brand value $\mathrm {N}\left( {\upalpha }_{\mathrm {ij,t}}, {\upsigma }_{\mathrm {ij,t}}^{\mathrm {2}} \right)$, with:

A.8 $$\begin{aligned} {\upalpha }_{\mathrm {ij,t+1}}=\frac{\frac{{\upalpha }_{\mathrm {ij,t}}}{{\upsigma }_{\mathrm {ij,t}}^{\mathrm {2}}}\mathrm {+}\frac{{\upeta }_{\mathrm {ijm,t}}}{{\upomega }_{\mathrm {ijm}}^{\mathrm {2}}}}{\frac{\mathrm {1}}{{\upsigma }_{\mathrm {ij,t}}^{\mathrm {2}}}\mathrm {+}\frac{\mathrm {1}}{{\upomega }_{\mathrm {ijm}}^{\mathrm {2}}}}\mathrm {,\, \quad }{\upsigma }_{\mathrm {ij,t}}^{\mathrm {2}}\mathrm {=\, }\frac{\mathrm {1}}{\frac{\mathrm {1}}{{\upsigma }_{\mathrm {ij,t}}^{\mathrm {2}}}\mathrm {+}\frac{\mathrm {1}}{{\upomega }_{\mathrm {ijm}}^{\mathrm {2}}}}\,. \end{aligned}$$

The prior mean ${\upalpha }_{\mathrm {ij,0}}$ is a linear function of brand ownership, the prior variance ${\upsigma }_{\mathrm {ij,0}}^{\mathrm {2}}$ is a function of brand familiarity, and the variance of the signal ${\upomega }_{\mathrm {ijm}}^{\mathrm {2}}$ is a function of experience with attribute m. The expected choice utility of a brand equals:

A.9 $$\begin{aligned} \mathrm {U}_{\mathrm {ij,t\, (c)}}\mathrm {=-exp}\left( \mathrm {-r\cdot }{\upalpha }_{\mathrm {ij,t+1}}\mathrm {+}\frac{\mathrm {r}^{\mathrm {2}}}{\mathrm {2}}{\upsigma }_{\mathrm {ij,t}}^{\mathrm {2}} \right) , \end{aligned}$$

reflecting risk aversion with a risk coefficient r, by which a higher posterior uncertainty about a brand, ${\upsigma }_{\mathrm {ij,t}}^{\mathrm {2}}$, decreases its utility. The inclusion of uncertainty as a function of risk aversion in equation (A.9) prioritizes eye-movements toward attributes that have received limited attention. Furthermore, like Yang et al. (2015), consumers are assumed to be one-step ahead forward looking, such that the utility derived from searching attribute m of product j is the maximum utility derived from choosing one of the alternatives at time t $+$ 1:

A.10 $$\begin{aligned} \mathrm {U}_{\mathrm {ij,t\, (s)}}={\mathrm {max}}\left\{ \mathrm {U}_{\mathrm {ij,t+1\, (c)}}, \mathrm {U}_{\mathrm {ik,t\, (c)}} \right\} \mathrm {-}\mathrm {c}_{\mathrm {ij}}. \end{aligned}$$

Equation (A.10) provides the expected value of choosing j at t$+$1 after an additional search of j (the first term) minus the search costs $\mathrm {c}_{\mathrm {ij}}$, or choosing any other option k at time t (the second term) minus the search costs. The latter are parameterized as a function of the vertical and horizontal saccade length, and fixations on white space.

### Satisficing Model of Search and Choice

Stüttgen et al. (2012) extend the model by van der Lans et al. (2008a) to a three-state HMM of satisficing choice, where $\mathrm {z}_{\mathrm {i,t}}$ indicates the state of individual i at fixation t. The model assumes that a consumer assigns the status satisfactory ($\mathrm {S}_{\mathrm {ij,t}})$, unsatisfactory ($\mathrm {U}_{\mathrm {ij,t}})$, or undetermined ($\mathrm {D}_{\mathrm {ij,t}})$ to each product at fixation t. If a product has not been fixated, its status is undetermined. To determine whether a product is satisfactory, a consumer needs to fixate all attributes m of that product and the value of each attribute needs to be acceptable, captured by ${\uppsi }_{\mathrm {im}}{\in }\left\{ 0,1 \right\} {,\,}$ which follows a Bernoulli distribution. Thus:

A.11 $$\begin{aligned} \mathrm {S}_{\mathrm {ij,t}}=\prod \limits _{\mathrm {m=1}}^{\mathrm {M}_{\mathrm {i}}} {\uppsi }_{\mathrm {im}}, \mathrm {U}_{\mathrm {ij,t}}={\mathop {\hbox {max}}\limits _{\mathrm{m}}}{(1-{\uppsi }_{\mathrm {im}}\mathrm {)}},\hbox { and }\mathrm {D}_{\mathrm {ij,t}}\mathrm {=1-}\mathrm {S}_{\mathrm {ij,t}}\mathrm {-}\mathrm {U}_{\mathrm {ij,t}}. \end{aligned}$$

The first two states ($\mathrm {z}_{\mathrm {i,t}}{\in }\left\{ g,l \right\} )$ in the HMM reflect the search process. In the global state (g), the decisions to fixate an ROI h follow a multinomial logit model with probabilities that are functions of the current status $\left\{ \mathrm {S}_{\mathrm {ij,t}}{\mathrm {,U}}_{\mathrm {ij,t}} \right\}$ of all products j and the size of ROI h. In the local state (l), the probabilities of fixating an ROI h are a function of the previously fixated ROI and of the ROIs bordering on it (reflecting local and re-fixations) captured via a vector of indicators $\mathrm {L}_{\mathrm {t}}\mathrm {(h)}$, in addition to a by-attribute strategy, captured via a vector of indicators $\mathrm {S}_{\mathrm {t}}\mathrm {(h)}$. The fixation probabilities in the local (${\upvarphi }_{\mathrm {l,t}}\mathrm {)}$ and global states (${\upvarphi }_{\mathrm {g,t}}\mathrm {)}$, with ${\upbeta }_{\mathrm {i,\cdot ,\cdot }}$ individual-level parameters to be estimated, are:

A.12 $$\begin{aligned}&{\upvarphi }_{\mathrm {g,t}}\mathrm {(h)}\propto \mathrm {r(h)exp}({\upbeta }_{\mathrm {i,g,0}}\mathrm {+}{{\upbeta }_{\mathrm {i,g,1}}\mathrm {S}}_{\mathrm {ij,t}}{\mathrm {+}{\upbeta }_{\mathrm {i,g,2}}\mathrm {U}}_{\mathrm {ij,t}}\mathrm {)} \end{aligned}$$

A.13 $$\begin{aligned}&{\upvarphi }_{\mathrm {l,t}}\mathrm {(h)}\propto \mathrm {exp}({\upbeta }_{\mathrm {i,l,0}}\mathrm + {{\upbeta }_{\mathrm {i,l,1}}\mathrm {S}}_{\mathrm {ij,t}}{\mathrm + {\upbeta }_{\mathrm {i,l,2}}\mathrm {U}}_{\mathrm {ij,t}}{\mathrm + {\upbeta }_{\mathrm {i,l,3}}^{\mathrm {'}}\mathrm {L}}_{\mathrm {t}}\mathrm {(h)}{\mathrm + {\upbeta }_{\mathrm {i,l,4}}^{\mathrm {'}}\mathrm {S}}_{\mathrm {t}}\mathrm {(h))} \end{aligned}$$

In the termination state ($\mathrm {w})$, the consumer chooses a product j that is satisfactory. Because the majority of participants continued searching after fully evaluating their final choice (which violates pure satisficing), the model assumes a verification stage to capture this. Therefore, it is possible that multiple products are satisfactory, leading to choice probabilities of satisfactory products that are equal to $\mathrm {p}\left( {\mathrm {y}_{\mathrm {ij}}\mathrm {=1}}\vert {\mathrm {S}_{\mathrm {ij,T}}\mathrm {=1}}\right) =1 / S$, while unsatisfactory products have a choice probability that equals zero: $\mathrm {p}\left( {\mathrm {y}_{\mathrm {ij}}\mathrm {=1}}\vert {\mathrm {U}_{\mathrm {ij,T}}\mathrm {=1}}\right) \mathrm {=0,}$ with *S* a normalizing constant. Finally, at odds with the theory of satisficing, some participants selected undetermined products, which was accommodated by a probability close to zero $\mathrm {p}\left( \mathrm {y}_{\mathrm {ij}} =1\big | \mathrm {D}_{\mathrm {ij,T}}=1\right) {\propto }{\epsilon } / \mathcal {S}with ({\epsilon }\sim Beta)$. Switching probabilities between the three states represented by $\mathrm {z}_{\mathrm {i,t}}$ are proportional to:

A.14 $$\begin{aligned} \mathrm {p}\left( \mathrm {z}_{\mathrm {i,t}} \right) {\propto }\left\{ {\begin{array}{*{20}l} \mathrm {exp}\left( {\uplambda }_{\mathrm {z}_{\mathrm {i,t}}\mathrm {0}}\mathrm {+}{\uplambda }_{\mathrm {z}_{\mathrm {i,t}}\mathrm {1}}\mathrm {I}\left( \mathrm {z}_{\mathrm {i,t-1}}\mathrm {=1} \right) \mathrm {+}{\uplambda }_{\mathrm {z}_{\mathrm {i,t}}\mathrm {2}}\mathrm {S}_{\mathrm {i}\mathrm {f}_{\mathrm {i,t-1}}\mathrm {,t-1}}\mathrm {+}{\uplambda }_{\mathrm {z}_{\mathrm {i,t}}\mathrm {3}}\mathrm {U}_{\mathrm {i}\mathrm {f}_{\mathrm {i,t-1}}\mathrm {,t-1}}\right) \quad &{}{} \mathrm {if}\, \mathrm {z}_{\mathrm {i,t}}\in \left\{ 1,2 \right\} \quad \quad \\ \mathrm {exp}\left( {\uplambda }_{\mathrm {z}_{\mathrm {i,t}}\mathrm {0}}\mathrm {+}{\uplambda }_{\mathrm {z}_{\mathrm {i,t}}\mathrm {1}}\mathrm {I}\left( \mathrm {z}_{\mathrm {i,t-1}}\mathrm {=1} \right) \mathrm {+}{\uplambda }_{\mathrm {z}_{\mathrm {i,t}}\mathrm {2}}\mathrm {N}_{\mathrm {i,t-1}}\mathrm {+}{\uplambda }_{\mathrm {z}_{\mathrm {i,t}}\mathrm {3}}{\mathop {\text{ max }}\limits _{\mathrm {j}}} {\mathrm {(}\mathrm {S}_{\mathrm {ij,t-1}}\mathrm {)}}\right) &{}{} \mathrm {if }\quad \mathrm {z}_{\mathrm {i,t}}=3\\ \end{array} } \right. \end{aligned}$$

In equation (A.14), ${\uplambda }_{\mathrm {z}_{\mathrm {i,t}}\mathrm {0}}$ captures the baseline switching probability to state $\mathrm {z}_{\mathrm {i,t}}\mathrm {.} \quad {\uplambda }_{\mathrm {z}_{\mathrm {i,t}}\mathrm {1}}$ captures the dependence of that switching probability on the previous state $\mathrm {z}_{\mathrm {i,t-1}}$. Further, switching to the global and local states depends on whether the previously fixated product $\mathrm {f}_{\mathrm {i,t-1}}$ is satisfactory or unsatisfactory, while switching to the termination states depends on the number of fixations $\mathrm {N}_{\mathrm {i,t-1}}$, and whether any of the choice alternatives is currently determined satisfactory.
